# Association of promoter methylation statuses of congenital heart defect candidate genes with Tetralogy of Fallot

**DOI:** 10.1186/1479-5876-12-31

**Published:** 2014-01-31

**Authors:** Wei Sheng, Yanyan Qian, Ping Zhang, Yao Wu, Huijun Wang, Xiaojing Ma, Long Chen, Duan Ma, Guoying Huang

**Affiliations:** 1 Children Hospital of Fudan University, Shanghai 201102, China; 2 Department of Biochemistry and Molecular Biology, Key Laboratory of Molecular Medicine, Ministry of Education, Institutes of Biomedical Sciences, Shanghai Medical College, Fudan University, Shanghai 200032, China; 3 Shanghai Key Laboratory of Birth Defects, Shanghai 201102, China; 4 Department of Forensic Medicine, Fudan University, Shanghai 200032, China

**Keywords:** DNA methylation, Congenital heart defect candidate genes, Tetralogy of Fallot

## Abstract

**Background:**

Although a lower methylation level of whole genome has been demonstrated in Tetralogy of Fallot (TOF) patients, little is known regarding changes in specific gene DNA methylation profiles and the possible associations with TOF. In current study, the promoter methylation statuses of congenital heart defect (CHD) candidate genes were measured in order to further understand epigenetic mechanisms that may play a role in the development of TOF.

**Methods:**

The methylation levels of CHD candidate genes were measured using the Sequenom MassARRAY platform. QRT-PCR was used to analyze the mRNA levels of CHD candidate genes in the right ventricular myocardium of TOF cases and normal controls.

**Results:**

Methylation status analysis was performed on the promoter regions of 71 CHD candidate genes (113 amplicons). We found significant differences in methylation status, between TOF cases and controls, in 26 amplicons (26 genes) (*p* < 0.05). Of the 26 amplicons, 17 were up regulated and 9 were down regulated. Additionally, 14 of them were located in the CpG islands, 7 were located in the CpG island shores, and 5 were covering the regions near the transcription start site (TSS). The methylation status was subsequently confirmed and mRNA levels were measured for 7 represented candidate genes, including *EGFR, EVC2, NFATC2, NR2F2, TBX5, CFC1B* and *GJA5*. The methylation values of *EGFR*, *EVC2, TBX5* and *CFC1B* were significantly correlated with their mRNA levels (*p* < 0.05).

**Conclusions:**

Aberrant promoter methylation statuses of CHD candidate genes presented in TOF cases may contribute to the TOF development and have potential prognostic and therapeutic significance for TOF disease.

## Background

A congenital heart defect (CHD) is an abnormality in the structure and great vessels of the heart, which is present at birth. About 19–75 people per 1000 are born with CHD each year, depending on which types of defects are included [1] and the incidence is higher if stillbirths were included [2]. Tetralogy of Fallot (TOF) is the most common complex congenital heart disease [3], accounting for 10% of all congenital heart diseases [4]. The four distinct anatomical features that characterize TOF are pulmonary outflow tract obstruction, ventricular septal defect, overriding aortic root and right ventricular hypertrophy [5]. TOF is considered to be a neural crest cell and/or second heart field related conotruncal heart defects that occur during embryonic development [6]. Although advances in surgical techniques, cardiopulmonary bypass and postoperative care in the last few decades have brought us today to where survival after complete repair is greater than 98% in large congenital heart surgery programs [7] and have improved the life quality of TOF patients to an excellent condition, late sudden cardiac death remains a persistent risk, with an estimated incidence of 0.5% to 6% [8], however, the exact etiology of TOF remains unclear. Some studies have reported that some of these defects related to TOF may be associated with chromosomal aneuploidy, including trisomies 13, 18 and 21 [9]. In addition to chromosomal rearrangements, mutations in a number of genes have also been found to be associated with TOF. A variety of gene insertion/deletion has been detected in patients with TOF. These genes include *JAG1*[10,11], *NKX2-5* and *GATA4*[12,13] etc. However, a single gene mutation or deletion can be identified only in a small percentage of cases [14]. Thus, we hypothesize that aberrant epigenetic regulation may be another important factor associated with TOF. Epigenetics refers to the heritable changes in genome function that occur without a change in the primary DNA sequence. These are characterized by covalent modifications of cytosine bases and histones, and changes in the positioning of nucleosomes [15]. Currently, the most widely studied epigenetic modification in humans is DNA methylation. DNA methylation can control the transcriptional activity of genes by various mechanisms, which occurs almost exclusively in the context of CpG dinucleotides [16]. The CpG dinucleotides tend to cluster in regions called CpG islands. Studies have proven that over 60% of human gene promoters are overlapped with CpG islands and are usually unmethylated in normal cells [17]. However, recent work on colon cancer has demonstrated that DNA methylation not only occurs at CpG islands, but also takes place in regions of lower CpG density that lie in close proximity (~2 kb) to the CpG islands, which is known as the CpG island shore [18]. The CpG island shores are the most enriched with functional CpG sites and have variable methylation, which are closely associated with transcriptional regulation. Most of the tissue-specific DNA methylation seems to occur, not at CpG islands, but at CpG island shores [19,20].

Methylation within gene promoters and CpG-dense sequences (CpG islands) have the highest functional relevance to gene expression control and the aberrant methylation changes contribute to many diseases [15]. The methylation patterns of multiple genes can provide different types of useful information about cancer cells [21]. The CpG island methylator phenotype, referring to the concurrent methylation of multiple genes, is a useful marker for tumor progression and has been reported in hepatocellular carcinoma [22]. However, although a lower methylation level of global genome has been demonstrated in TOF patients [23], little is known about gene-specific DNA methylation changes in patients with TOF.

In this study, we selected 71 CHD candidate genes, based on the evidences of methylation microarray performed previously [24], transcriptional studies, mouse models and their close association with the heart development, to explore their promoter DNA methylation changes and their association with TOF development.

## Materials and methods

### Patients and controls

The TOF cases were recruited from the Children’s Hospital of the Fudan University, Shanghai, China. Cardiovascular diagnosis was obtained by echocardiography. All TOF cases were tested for chromosome anomalies by karyotype and 22q11 deletion by fluorescent in situ hybridization (FISH) as previously described [25]. Patients with trisomy 21, 22q11 deletion, other chromosomal anomalies, or extracardiac major or minor associated anomalies were discarded in this study. A total of 41 TOF cases were recruited, including 26 (63.4%) males and 15 (36.6%) females, ranging in age from 2.0 to 48.0 months [11.0 (7.0 - 24.0), median (interquartile range)].

The control group was comprised of necropsy specimens from normal subjects that had died as a result of an accident. Necropsy specimens were collected at the forensic medicine department of the Fudan University, Shanghai, China. Considering a delay with necropsy, the post mortem interval (PMI) for the control samples was as short as possible. Six age-matched normal controls were collected, including 4 (66.7%) males and 2 (33.3%) females, ranging in age from 6.0 to 54.0 months [15.0 (10.5 - 30.0), median (interquartile range)]. Anagraphical characteristics of the study subjects are summarized in Table 1.

**Table 1:** Anagraphical characteristics of TOF cases and normal controls

**Characteristic**	**TOF (N = 41) (months)**	**Control (N = 6) (months)**
Age [median (IQR)]	11.0 (7.0 – 24.0 )	15.0 (10.5 – 30.0)
Gender		
Male (%)	26 ( 63.4 )	4 ( 66.7 )
Female (%)	15 ( 36.6 )	2 ( 33.3 )

*IQR*, Interquartile range.

To exclude the tissue heterogeneity that may affect methylation levels, all heart tissue samples from TOF patients and controls were obtained from the right ventricular myocardial tissues and saved in storage solution (RNAlater®, AMBION, Inc., Austin, USA) immediately following surgical resection or necropsy and then stored in -80°C until use.

The Fudan University’ Ethics Committee approved this study. Written informed consents were obtained from the parents or relatives of all study subjects.

### DNA extraction and sodium bisulfite conversion

Genomic DNA was extracted from myocardial tissues using a QIAamp DNA Mini Kit (Qiagen, Hilden, Germany) according to the manufacturer’s instructions. The concentration and purity of the DNA were determined by absorbance at 260 and 280 nm using a NanoDrop™ 1000 Spectrophotometer (Thermo Scientific, Wilmington, DE, USA) and agarose gel. Sodium bisulfite modification was performed on the extracted DNA, using an EZ DNA Methylation Kit™ (Zymo Research, Orange, CA, USA) strictly following the manufacturer’s instructions. The bisulfite-converted DNA was re-suspended in 10 μl elution buffer and stored at -80°C until the samples were ready for analysis.

### MassARRAY quantitative methylation analysis

The Sequenom MassARRAY platform was used to perform the quantitative methylation analysis for the promoter regions of 71 CHD candidate genes. The methylation status of a detected pattern was then analyzed using Epityper software version 1.0 (Sequenom, San Diego, CA, USA). The promoter regions of the 71 CHD candidate genes were chosen using the website http://genome.ucsc.edu (shown in Additional file 1: Table S1). The 113 amplicons and PCR primers used in this system were designed using the website http://epidesigner.com (Additional file 1: Table S2).

All experiments were performed as described before [23]. Non-applicable readings and their corresponding sites were eliminated from analysis. The methylation level of the genes promoter regions is expressed as the percentage of methylated cytosines over the total of methylated and unmethylated cytosines.

### RNA extraction and quantitative RT-PCR

Total RNA was extracted from tested myocardial tissue samples using Trizol Reagent (Invitrogen, CA, USA) according to the manufacturer’s instructions. The extracted RNA quality and integrity were validated before use. RNA was reverse-transcribed using a PrimeScript RT reagent Kit with gDNA Eraser (Perfect Real Time) (TaKaRa, Japan) and the integrity of synthesized cDNA was confirmed using glyceraldehyde 3-phosphate dehydrogenase (GAPDH) as the endogenous control. Quantitative RT-PCR was performed in a 7900 real-time PCR system using SYBR Premix Ex Taq GC (Perfect Real Time) (TaKaRa, Japan) following the manufacturer’s instructions. Reactions were performed in triplicate and analyzed using an ABI 7900 Sequence Detection System (Applied Biosystems, Foster City, CA, USA). Relative expression levels were calculated according to the standard 2^-ΔΔCt^ method using beta-2 microglobulin (B2M) and the GAPDH gene as the endogenous control for normalization. Primer sequences used in QRT-PCR analysis are listed in Additional file 1: Table S3.

### Statistical analysis

The methylation status of the CHD candidate genes’ promoter regions was analyzed using GraphPad Prism (version 5.0; GraphPad Software Inc., San Diego, CA, USA) and SPSS (version 13.0; SPSS Inc., Chicago, IL, USA). A Mann–Whitney test was used to evaluate the significance of any differences between TOF cases and normal controls. Spearman’s rank correlation was used to examine the relationship between two continuous variables. All statistical analyses were 2-sided and *P <* 0.05 was considered statistically significant.

## Results

### Methylation status analyses of the promoter region of 71 CHD candidate genes in TOF cases and controls

To comprehensively understand the promoter regions of CHD candidate genes that were analyzed, we focused on the region covering 2000 bp + 200 bases relative to transcription start sites (TSS). Based on the information regarding the promoter region in each of 71 candidate genes, we designed a total of 113 amplicons, of which 72 covered the CpG island (64%), 24 covered the CpG island shore (21%) and 17 were not associated with CpG island (15%) (shown in Table 2). Using the Sequenom MassARRAY platform, the methylation levels in the promoter regions of these genes were detected in 10 TOF cases and 6 controls for the first round of screening. Prior to analysis, we performed strict quality control to remove any potentially unreliable measurements, such as low mass, high mass, and silent peak overlap CpG units. The CpG units that failed to produce data in more than 30% of samples (unreliable CpG units) and samples that were missing more than 30% of the data points (unreliable samples) were discarded [26]. The mean and median methylation levels for the 113 amplicons (71 candidate genes) in the TOF cases and controls are shown in Additional file 1: Table S4.

**Table 2:** Information on the promoter region of analyzed candidate genes

	**Amplicon**	**Gene**
In total	113	71
CpGI	72	50^a^
CpGI shore	24	23^b^
no CpG	17	12

*Note:*^a^and ^b^have overlapped genes.

The results indicated that the median methylation values in 26 of 113 amplicons (26 genes) were significantly different in TOF cases compared with controls (shown in Additional file 1: Table S5, *p <* 0.05). The methylation levels were up regulated in 17 genes, including *CFC1B, DVL2, EGFR, EDNRA, EVC2, GJA5, HAND1, HAS2, HSPG2, MED13L, NFATC1, NKX2-5, NFATC2, PAX3, TBX5, TEK* and *ZFPM2*. Whereas, the methylation levels was down regulated in 9 genes, including *EDN1, HOXA3, MYH6, NR2F2, NRG1, NRP1, PDGFRA, SLC2A10,* and *TBX20*. There were 14 amplicons (14 genes: *DVL2, EDN1, EGFR, EVC2, HAND1, HAS2, HSPG2, NKX2-5, NRG1, NFATC2, PAX3, PDGFRA, SLC2A10, TBX20*), located in the CpG islands; 7 amplicons (7 genes: *EDNRA, MED13L, NFATC1, NR2F2, NRP1, TBX5, ZFPM2*), were located in the CpG island shore, and 5 amplicons (5 genes: *CFC1B, GJA5, HOXA3, MYH6 ,TEK*), covered the region near transcription start site (TSS).

### Validation of the methylation statuses of seven candidate genes in TOF cases and controls

On the basis of the data analyzed above, 7 CHD candidate genes, including *EGFR, EVC2, NFATC2, NR2F2, TBX5, CFC1B* and *GJA5,* were chosen and validated for their methylation levels in the samples from 41 patients with TOF. These genes were selected because they function as key factors in the development of heart as well as showing significant differences in methylation status between TOF cases and controls. Moreover, the amplicon fragments measured in these genes were distributed in different regions of the promoter: *EGFR, EVC2* and *NFATC2* were associated with the CpG islands, *NR2F2* and *TBX5* with the CpG island shores and *CFC1B* and *GJA5* were in the regions near the TSS.

**Figure 1:**
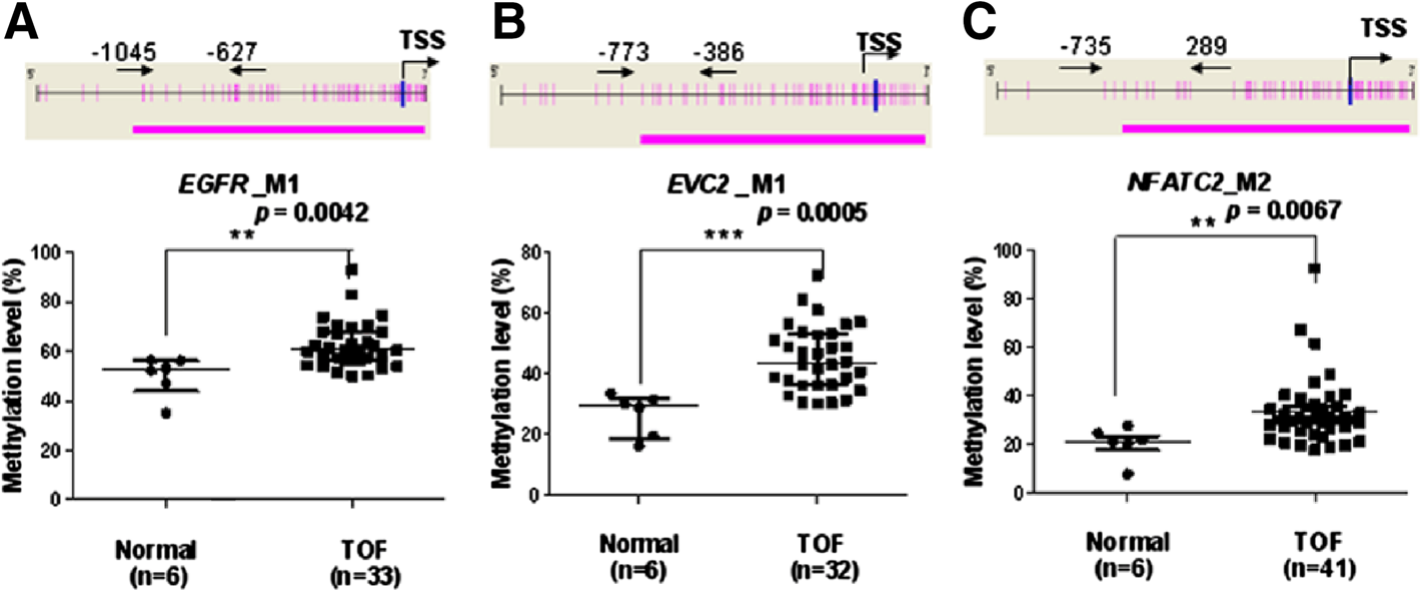
**Median methylation levels in the promoter CpG islands of candidate genes of TOF cases and controls. (A)***EGFR_*M1 median methylation level; **(B)***EVC2_*M1 median methylation level; **(C)***NFATC2_*M2 median methylation level. TSS, Transcription Start Sites; red vertical line, CpG sites; red thick bars, CpG islands; M, MassARRAY amplicon; The region between two arrows, target amplicon. *P < 0.05, **P < 0.01, ***P < 0.001 (Mann–Whitney test).

As shown in Table 3, the significant differences for methylation values of the 7 genes were confirmed in a larger number of samples. In the CpG island region of gene promoter in TOF cases, the methylation levels of the following genes were significantly up-regulated: *EGFR* (52.75% *vs* 60.50%, median, *p =* 0.0042, Figure 1A), *EVC2* (29.63% *vs* 43.88%, median, *p =* 0.0005, Figure 1B), and *NFATC2* (21.80% *vs* 30.29%, median, *p = *0.0067, Figure 1C). The concurrent higher methylation of *EGFR, EVC2* and *NFATC2* might represent a CpG island methylator phenotype in TOF development.

**Table 3:** The median methylation values for 7 CHD candidate genes showing significant differences between TOF cases and controls

**Gene**	**Gene bank accession**	**Gene amplicon**	**Control, median (IQR); N**	**TOF, median (IQR); N**	***P *** **value**	**Position**
*EGFR*	NM_201282	*EGFR* _M1	52.75(44.00-56.35);N = 6	60.50(56.10-68.08);N = 33	0.0042**	CpGI
*EVC2*	NM_001166136	*EVC2* _M1	29.63(18.84-32.25);N = 6	43.88(36.44-53.28);N = 32	0.0005***	CpGI
*NFATC2*	NM_001136021	*NFATC2*_M2	21.80(17.50-25.53);N = 6	30.29(26.06-36.57);N = 41	0.0067**	CpGI
*NR2F2*	NM_021005	*NR2F2*	62.50(54.35-68.30);N = 6	43.40(33.90-53.10);N = 41	0.0161*	CpGI shore
*TBX5*	NM_080717	*TBX5*_M1	42.04(32.97-54.69);N = 6	57.13(50.69-67.31);N = 37	0.0207*	CpGI shore
*CFC1B*	NM_001079530	*CFC1B*_M2	48.16(40.36-59.25);N = 6	63.50(53.57-80.60);N = 39	0.0178*	TSS
*GJA5*	NM_005266	*GJA5*	32.83(29.00-36.08);N = 6	44.00(34.67-53.92);N = 36	0.0138*	TSS

*P < 0.05, **P < 0.01, ***P < 0.001 (Mann–Whitney test), *IQR*, Interquartile range.

**Figure 2:**
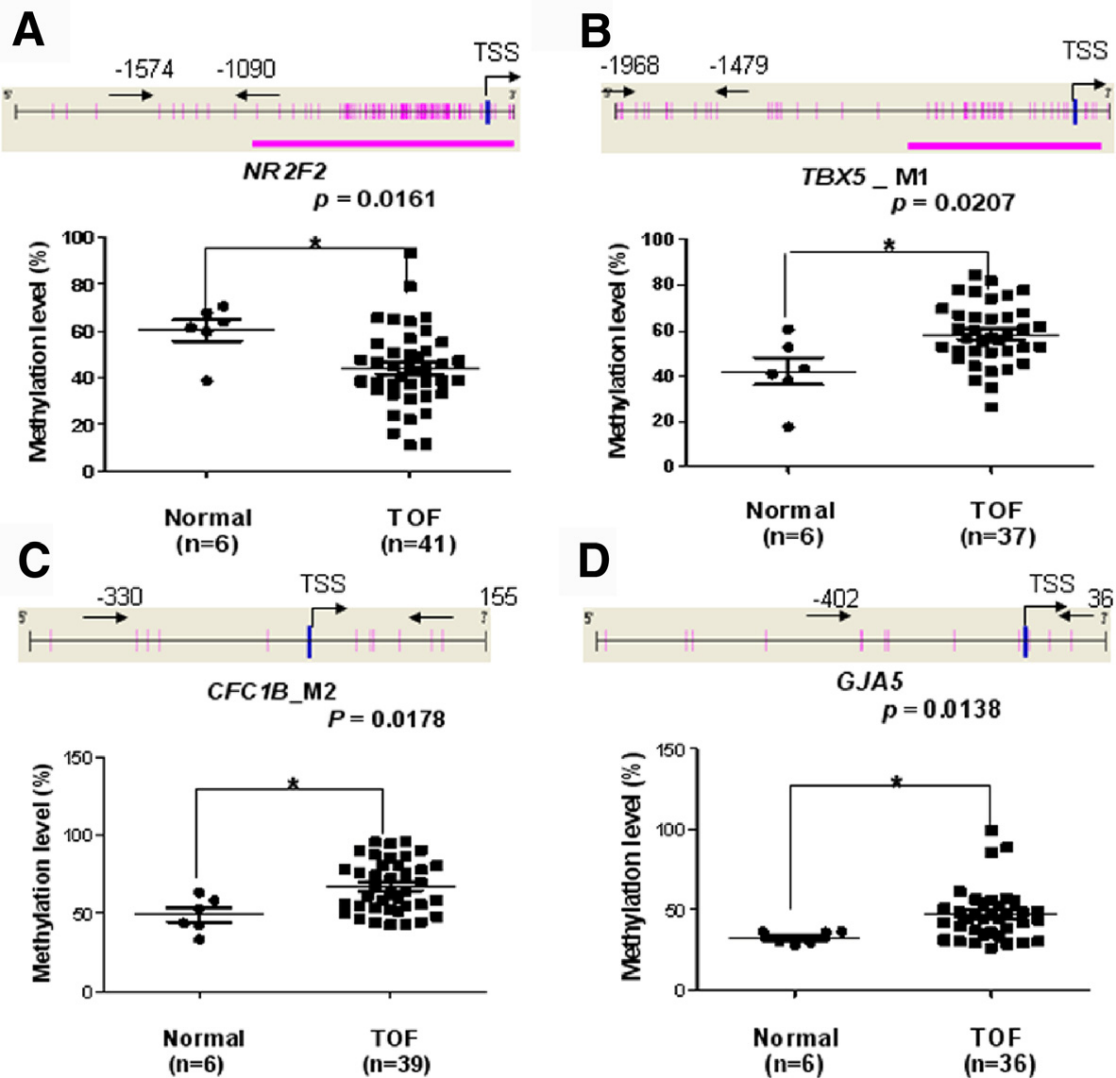
**Median methylation levels in the promoter CpG island shores and the promoter region located outside of the CpG island but near the TSS between TOF cases and controls. (A)***NR2F2* median methylation level; **(B)***TBX5* median methylation level; **(C)***CFC1B* median methylation level; **(D)***GJA5* median methylation level; TSS, Transcription Start Sites; red vertical line, CpG sites; red thick bars, CpG islands; The region between two arrows, target amplicon. *P < 0.05, **P < 0.01, ***P < 0.001 (Mann–Whitney test).

In the CpG island shore region of gene promoter in TOF cases, the methylation level of *NR2F2* was significantly down regulated (62.50% *vs* 43.40%, median, *p =* 0.0161, Figure 2A), though the methylation level of *TBX5* was higher (42.04% *vs* 57.13%, median, *p =* 0.0207, Figure 2B) in TOF cases.

Furthermore, in the TSS promoter region of gene in TOF cases, the methylation levels of *CFC1B_*M2 and *GJA5* were significantly up regulated (48.16% *vs* 63.50%, median, *p =* 0.0178, Figure 2C; 32.83% *vs* 44.00%, median, *p =* 0.0138, Figure 2D; respectively) compared to controls.

### mRNA expression levels of seven CHD candidate genes in TOF cases and controls

QRT-PCR was performed to determine the mRNA expression levels of the 7 CHD candidate genes in TOF cases and normal controls. As shown in Table 4, the mRNA levels of the 7 candidate genes were significantly lower in TOF cases than in controls (*p <* 0.05).

**Table 4:** Median mRNA levels for 7 candidate genes in TOF cases and controls

**Gene**	**Gene bank accession**	**Control, median (IQR), N**	**TOF, median (IQR), N**	**mRNA fold change**	***P *** **value**
*EGFR*	NM_201282	3.98 (1.84 – 6.93); N = 6	0.61 (0.46 – 0.75); N = 33	0.15	0.0003***
*EVC2*	NM_001166136	3.95 (1.50 – 6.21); N = 6	0.96 (0.79 – 1.56); N = 32	0.24	0.0033**
*NFATC2*	NM_001136021	7.72 (4.60 – 11.50); N = 6	1.39 (0.97 – 1.88); N = 41	0.18	0.0043**
*NR2F2*	NM_021005	3.27 (1.34 – 4.88); N = 6	1.12 (0.70 – 1.30); N = 41	0.34	0.0039**
*TBX5*	NM_080717	1.49 (1.35 – 4.42);N = 6	0.41 (0.19 – 0.67); N = 37	0.28	0.0013**
*CFC1B*	NM_001079530	2.20 (1.57 – 7.50); N = 6	0.66 (0.31 – 1.07); N = 39	0.30	0.0016**
*GJA5*	NM_005266	0.77 (0.34 – 1.06); N = 6	0.19 (0.16 – 0.32); N = 36	0.25	0.0053**

**P < 0.01, ***P < 0.001 (Mann–Whitney test), *IQR*, Interquartile range.

### Correlations between methylation statuses of seven CHD candidate genes and their respective mRNA levels

**Figure 3:**
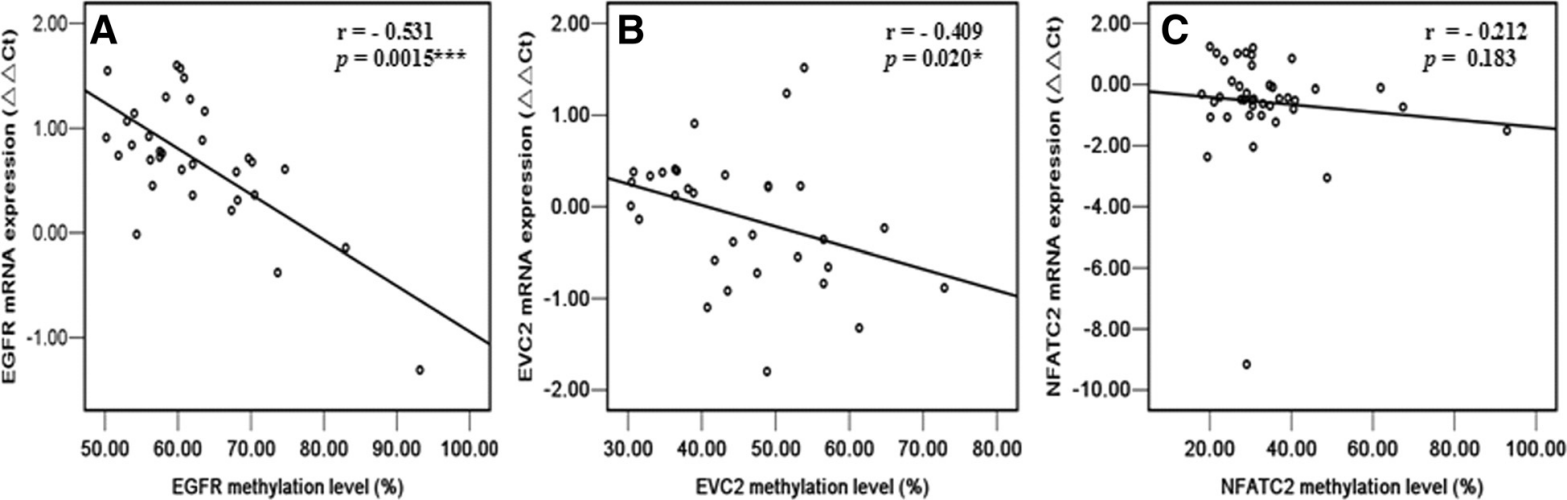
**Correlation of methylation status in the promoter CpG islands of candidate genes with their mRNA levels in TOF cases. (A)***EGFR* gene; **(B)***EVC2* gene; **(C)***NFATC2* gene. *P < 0.05, **P < 0.01, ***P < 0.001. (Spearman’s rank correlation test).

A correlation analysis was then done in the TOF cases to identify whether there was any relationship between the methylation statuses and mRNA levels of 7 CHD candidate genes. In the promoter CpG island region, two significant associations were found between the methylation statuses and mRNA levels for *EGFR* (r = - 0.531, *p =* 0.0015, Figure 3A) and *EVC2* (r = - 0.409, *p =* 0.020, Figure 3B). No significant correlation was observed between the methylation status and mRNA level for *NFATC2* (r = - 0.212, *p =* 0.183, Figure 3C).

**Figure 4:**
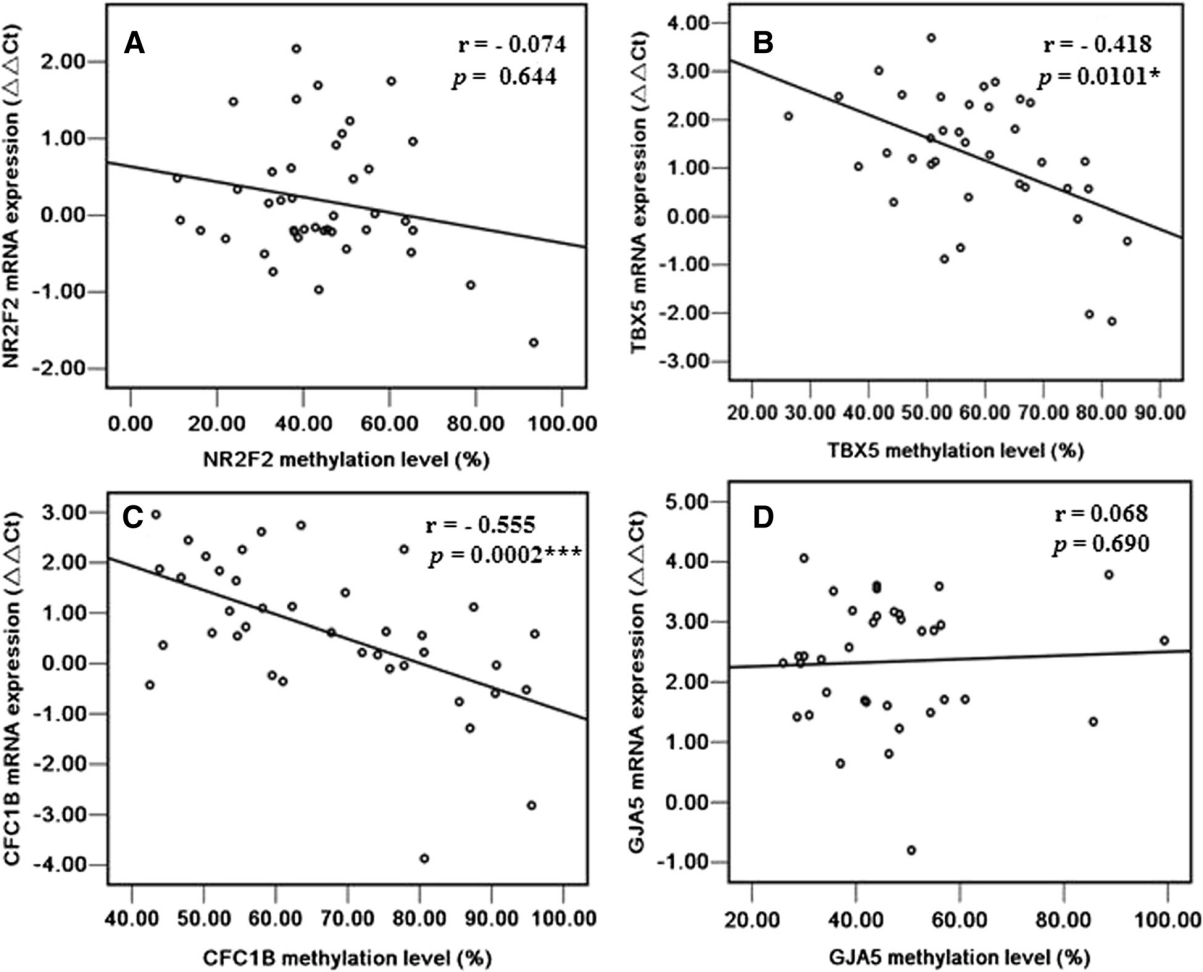
**Correlation of methylation status in the promoter CpG island shore and the promoter region near TSS of candidate genes with their mRNA levels in TOF cases. (A)***NR2F2* gene; **(B)***TBX5* gene; **(C)***CFC1B* gene; **(D)**GJA5 gene.*P < 0.05, **P < 0.01, ***P < 0.001. (Spearman’s rank correlation test).

In the CpG island shore region, no significant correlation was observed between the methylation status and mRNA level of *NR2F2* (r = - 0.074, *p =* 0.644, Figure 4A). Interestingly, the methylation status of *TBX5* was negatively correlated with its mRNA expression level (r = - 0.418, *p =* 0.0101, Figure 4B).

A strong correlation was found between the methylation level in the promoter region near the TSS of *CFC1B* and its mRNA level (r = - 0.555, *p =* 0.0002, Figure 4C). However, the methylation status of *GJA5* was not significantly associated with its mRNA level (r = 0.068, *p =* 0.690, Figure 4D).

## Discussion

DNA methylation constitutes an important epigenetic regulation mechanism in many eukaryotes [27]. In previous study, we have demonstrated the hypomethylation of LINE-1, which can serve as an indicator of global DNA methylation [28], in the myocardial tissue of TOF patients [23]. In the present study, quantitative methylation analysis was performed on the promoter region of 71 CHD candidate genes in the myocardial tissue of TOF cases and normal controls using the Sequenom MassARRAY platform. This system combines base-specific enzymatic cleavage with MALDI-TOF mass spectrometry and creates a highly accurate, sensitive, and high-throughput method for the quantitative analysis of DNA methylation at CpG units [29]. The robustness of this approach for quantifying methylated and unmethylated DNA has been confirmed by the Sequenom group [30]. We found that 26 of the 71 candidate genes showed significant differences in the methylation levels when comparing TOF cases to controls (*p* < 0.05). In which, 17 genes were up regulation and 9 genes were down regulation. DNA methylation analysis for multiple genes in TOF cases enables us to reveal the complex etiology of CHD from a novel aspect and provides potential development of new treatments for TOF disease.

The methylation statuses at different regions of the gene promoter may have different effects on the gene’s activities. The 26 genes showing significant differences in the methylation levels can be grouped to three categories according to the location of amplicons in the gene promoter. Fourteen amplicons (14 genes) were located in the CpG islands, 7 amplicons (7 genes) were located in the CpG island shore, and 5 amplicons (5 genes) were covering the region near the TSS.

Seven CHD candidate genes, including *EGFR, EVC2, NFATC2, NR2F2, TBX5, CFC1B* and *GJA5*, were chosen for further validation in a larger number of TOF cases because of their nominally significant differences in methylation levels and the important roles they play in the development of the heart. *EGFR*, a receptor tyrosine kinase in the *ErbB* family, activates several signaling cascades that convert extra-cellular cues into appropriate cellular responses and has been demonstrated to be associated with the congenital left ventricular outflow tract obstruction [31]. *EVC2* plays a critical role in bone formation and skeletal development and mutations in *EVC2* are associated with Ellis van Creveld syndrome in which 50-60% of congenital heart defects occur [32]. *NFATC2* is a DNA binding protein with a REL-homology region (RHR) and an NFAT-homology region (NHR). It plays a central role in inducing gene transcription during the immune response. Bourajjaj M. et all, 2008, have shown that *NFATC2* is a necessary mediator of calcineurin-dependent cardiac hypertrophy and heart failure [33]. *NR2F2* is a ligand inducible transcription factor that is involved in the regulation of many different genes and the deletion of *NR2F2* was considered to possibly contribute to congenital heart defects [34]. *TBX5* is a member of the T-box transcription factor family and plays an important role in heart development and the specification of limb identity, which is very well known associated with Holt Oram syndrome, a developmental disorder affecting the heart and upper limbs [35]. The regulatory variation in the TBX5 enhancer can lead to the same phenotype as a mutation in the gene. *CFC1B* is an important factor in embryo development. Mutations in this gene have been reported in Chinese children with congenital heart disease [36]. The *GJA5* gene encodes for the cardiac gap junction protein connexin 40. A variant in the carboxyl-terminus of connexin 40 alters GAP junctions and increases the risk for TOF [37].

Aberrant DNA methylation of CpG islands has been widely observed in human tumors and is associated with gene silencing when it occurs in promoter areas [38]. Multiple genes showing increased or decreased methylation simultaneously have been found in colorectal cancer [39] and in duodenal adenocarcinomas [40]. In the present study, we found significantly higher methylation levels in the promoter CpG islands of *EGFR, EVC2* and *NFATC2* in TOF cases compared with controls. The simultaneous higher methylation of *EGFR, EVC2* and *NFATC2* may represented a CpG island methylator phenotype in TOF development and provide useful clues as to the development of an epigenetic classification of the disease with prognostic and therapeutic potential. Moreover, *EGFR* and *EVC2* were found to have significant negative correlations between methylation values and respective mRNA expression levels (Figure 3A,B , *p <* 0.05), indicating that methylation changes at the CpG island region of the two genes may have influences on gene expression, though the exact control mechanism requires further study. Although the mRNA level of *NFATC*2 was significantly lower in TOF cases compared to the controls in our study (*p* < 0.05), no significant correlation between methylation level and mRNA expression was observed (Figure 3C, *p >* 0.05). We concluded that the analyzed methylation region at the promoter CpG island of *NFATC2* might not be involved in regulating the gene transcription.

A recent genome-wide analysis of DNA methylation showed that 76% of differential tissue methylation regions were not located in CpG islands, but in CpG island shore [18]. In this study, we found a decreased methylation level at the CpG island shore for *NR2F2* and an increased methylation level for *TBX5*. We cannot ascertain the factor that contributed to the lower methylation level of *NR2F2*. However, the methylation status of *NR2F2* were found not to be associated with its mRNA level, suggesting that methylation changes at the CpG island shore of *NR2F2* might not influence transcriptional activity. Further studies are needed to explore whether the methylation changes in the other region of *NR2F2* promoter influences its mRNA level. Interestingly, the methylation status of the *TBX5* gene was significantly negatively correlated with its mRNA level, indicating that increased methylation level at the CpG island shore of *TBX5* may inhibit this gene transcriptional activity.

DNA methylation can directly inhibit transcription by precluding the recruitment of DNA binding proteins from their target sites [41]. Cao et al. found that CpG site-specific methylation can alter binding affinities of specific transcription factors, which can differentially activate or repress transcription [42]. Consistent with this, Tihomira D et al. have reported that, although methylation of all CpG sites resulted in the silencing of *EphA5* promoter activity, lower levels of methylation resulted in differential activation or repression of *EphA5* promoter activity, depending on the sites methylated [43]. In the current study, the methylation levels at the promoter region near the TSS of *CFC1B* and *GJA5* were significantly higher in TOF cases compared to normal controls. In addition, other than *GJA5*, one significant negative correlation was observed between the methylation status and mRNA level of *CFC1B*, indicating that the methylation change at the promoter region near the TSS of *CFC1B* may play an important role in regulating the gene transcriptional activity. How the altered methylation statuses of *CFC1B* influence mRNA level requires further study.

However, in the present study, we have not examined the protein levels of these genes in the myocardial tissue because of the limited samples. The potential alterations of the protein levels and their associations with the transcription level would be explored in the further study.

## Conclusions

Our results suggest that multiple gene-specific DNA methylation changes at the promoter regions occurred in TOF cases and may be associated with TOF development. The concurrent higher methylation of *EGFR, EVC2* and *NFATC2* might constitute a CpG island methylator phenotype for TOF disease and provide useful cues to understand epigenetic mechanisms in the development of TOF. The methylation values of *EGFR*, *EVC2, TBX5* and *CFC1B* were significantly correlated with their respective mRNA levels (*p* < 0.05), indicating that aberrant methylation changes of specific genes may contributes to the development of TOF. Because heart tissue samples are difficult to collect from healthy controls and TOF patients, one limitation of this study is that we were unable to obtain enough complete matched samples. This could influence the accuracy of the methylation results. Also, we cannot be sure if the methylation patterns observed in the samples were an effect of or caused the TOF since the development of TOF long predated the measurement of methylation. Further studies with larger sample numbers are warranted to confirm our findings and to focus on whether aberrant methylation contributes to TOF development.

## Competing interests

The authors declare that they have no competing interests.

## Authors’ contributions

SW participated in study concept and design and coordination of the study, helped with the statistical analysis and drafted the manuscript. QY, WY, WH and MX participated in TOF sample acquisition and helped to draft the manuscript. ZP participated in normal control sample acquisition and helped to draft the manuscript. CL, MD and HG conceived of the study, and participated in its design and coordination and helped to draft the manuscript. All authors have read and approved the final manuscript.

## Supplementary Material

Additional file 1: Table S1 Congenital heart defect (CHD) candidate gene data. **Table S2.** Primer sequences, position, product length, and CpG site used for MassArray quantitative methylation analysis. **Table S3.** Primer sequences and product length for QPT-PCR analysis. **Table S4.** Mean and median methylation levels for 113 amplicons (71 candidate genes) in 10 TOF cases and 6 Controls. **Table S5.** The median methylation levels of 26 candidate genes showing significant difference in TOF cases and controls. Click here for file
