# Supplementary material for: Association of promoter methylation statuses of congenital heart defect candidate genes with Tetralogy of Fallot
Source: J Transl Med. 2014 Jan 31;12:31. doi: 10.1186/1479-5876-12-31 (PMC3915623; doi:10.1186/1479-5876-12-31)
Supplement: Additional file 1: Table S1 — Congenital heart defect (CHD) candidate gene data. Table S2. Primer sequences, position, product length, and CpG site used for MassArray quantitative methylation analysis. Table S3. Primer sequences and product length for QPT-PCR analysis. Table S4. Mean and median methylation levels for 113 amplicons (71 candidate genes) in 10 TOF cases and 6 Controls. Table S5. The median methylation levels of 26 candidate genes showing significant difference in TOF cases and controls. [file 1479-5876-12-31-S1.doc]

**Additional files**

**Additional file 1: Table S1．Congenital heart defect (CHD) candidate gene data**

|  | Gene name | Genomic  Location | Accession  numbers | Null  Phenotype | CpGI |
| --- | --- | --- | --- | --- | --- |
| 1 | ACVR1 (activin  A receptor, typeI) | chr2:158592958  -158731623 | NM_001111067 | Fibrodysplasia  ossificans progressiva (FOP), | CpGI |
| 2 | ACVR2B(activin  A receptor, typeIB) | chr3:38495790  -38534633 | NM_001106 | L Aterality defects,*  TGA, DORV, PTA | CpGI |
| 3 | ANGPT1  (Angiopoietin 1) | chr8:108261721  -108510283 | NM_001199859 | Acute myocardial infarction,  HypertensionPulmonary,  Cardiovascular Diseases | no CpGI |
| 4 | ASD1(atrial septal defect1,  Apx/shroom1) | [chr5:132157833 -132166590](http://genome.ucsc.edu/cgi-bin/hgTracks?position=chr5:132157833-132166590&hgsid=309151847&refGene=pack&hgFind.matches=NM_001172700,) | NM_001172700 | ASD | CpGI |
| 5 | BCOR(BCL6  corepressor) | chrX:39910499  -39956656 | NM_017745 | OFCD SYNDROME,  Mental Retardation,  Aberrant heart development | CpGI |
| 6 | BMP4(bone  morphogenetic protein 4 | chr14:54416455  -54423554 | NM_001202 | AVSD,congenital malformation,Hypertrophy | CpGI |
| 7 | CFC1B(cripto, FRL-1, cryptic  family 1B ) | chr2:131350353  -131357251 | NM_001079530 | AVSD, DORV,TOF,ASD | CpGI |
| 8 | CITED2(Cbp/p300-interacting transactivator, With  Glu/Asp-richcarboxy-terminal domain,2) | chr6:139693397  -139695785 | NM_006079 | ASD, VSD, DORV, PTA | CpGI |
| 9 | COX6A2(cytochrome  c oxidase subunit  VIa polypeptide 2) | chr16:31439052  -31439749 | NM_005205 | Aberrant heart development | CpGI |
| 10 | CRELD1(cysteine-rich  with EGF-like domains 1) | chr3:9975524  -9987097 | NM_015513 | AVSD*,atrioventricular  canal defects,Congenital  cardiac septal defects. | CpGI |
| 11 | DVL2(Dishevelled,  dsh homolog 2) | chr17:7128661  -7137863 | NM_004422 | PTA, TGA | CpGI |
| 12 | ECE1(endothelin  converting enzyme 1) | chr1:21545077  -21606042 | NM_001397 | IAA, VSD, DORV, PTA | no CpGI |
| 13 | EDN1(endothelin 1) | chr6:12290529  -12297427 | NM_001168319 | IAA, VSD | CpGI |
| 14 | EGFR(epidermal  growth factor receptor) | chr7:55086714  -55270769 | NM_201282 | AS, AI | CpGI |
| 15 | ELN(elastin) | chr7:73442427  -73484236 | NM_000501 | SVAS,Pulmonary Stenosis | CpGI |
| 16 | EDNRA(endothelin  receptor type A) | chr4:148402069  -148466106 | NM_001957 | IAA, VSD, DORV, PTA, TGA | CpGI |
| 17 | ERBB2(v-erb-b2  erythroblastic leukemia  viral oncogene homolog 2) | chr17:37856254  -37884915 | NM_004448 | Noncompaction,  aberrant trabeculation | CpGI |
| 18 | EVC(Ellis-van  Creveld syndrome protein ) | chr4:5712924  -5816031 | NM_153717 | DORV | CpGI |
| 19 | EVC2(Ellis van  Creveld syndrome 2) | chr4:5564146  -5711275 | NM_001166136 | DORV,POLYDACTYLY,  POSTAXIAL,  Heart Defects Congenital | CpGI |
| 20 | FGF8(Fibroblast  growth factor 8) | chr10:103529887  -103535759 | NM_033164 | DORV, PTA, ASD, VSD,  AV valve atresia,  arch hypoplasia | CpGI |
| 21 | FOXC1(Fkh1/fork  head box C1) | chr6:1610681  -1614129 | NM_001453 | Aortic arch abnormalities | CpGI |
| 22 | GATA4 (GATA  binding protein 4)* | chr8:11561717  -11617509 | NM_002052 | ASD,* VSD, cardia bifida,  ventral morphogenesis | CpGI |
| 23 | GJA1(Connexin43/gap  junction membrane  channel protein 1)* | chr6:121756745  -121770873 | NM_000165 | AVSD,RVOT obstruction,  aberrant coronary  patterning; heterotaxy | No CpGI |
| 24 | GJA5(Connexin43/gap  junction membrane  channel protein 1) | chr1:147228332  -147245484 | NM_005266 | VSD | No CpGI |
| 25 | HAND1(eHand/heart  and neural crest  derivatives expressed 1) | chr5:153854532  -153857824 | NM_004821 | Looping abnormality | CpGI |
| 26 | HAS2(Hyaluron  synthase 2) | chr8:122625271  -122653630 | NM_005328 | Absent AV cushions  and trabeculae | CpGI |
| 27 | HEY2(Hairy/enhancer-of-  split related with YRPW  motif protein 2) | chr6:126070732  -126082415 | NM_012259 | Heart Defects Congenital | CpGI |
| 28 | HOXA3(Hox-1.5/  homeobox A3) | chr7:27145809  -27159214 | NM_030661 | CT abnormalities,DGS | No CpGI |
| 29 | HSPG2(Perlecan/heparin  sulfate proteoglycan  of basement membrane) | chr1:22148737  -22263750 | NM_005529 | TGA/IVS, coronary  anomalies | CpGI |
| 30 | JAG1(Jagged 1)* | chr20:10618332  -10654694 | NM_000214 | PS, VSD, TOF,  Alagille’s syndrome* | CpGI |
| 31 | MED13L(Mediator of  RNA polymerase II  transcription subunit 13-like) | chr12:116396381  -116714991 | NM_015335 | transposition of  the great arteries | CpGI |
| 32 | MEF2C(Myocyte enhancer  factor 2C) | chr5:88014058  -88119744 | NM_001193348 | Cardiac Hypertrophy | No CpGI |
| 33 | MYH6(Myosin, heavy  polypeptide 6,  cardiac muscle, alpha) | chr14:23851199  -23877486 | NM_002471 | ardiomyopathy,  Heart Diseases,  MYOPATHY, CONGENITAL,ASD | No CpGI |
| 34 | MYH7(myosin, heavy chain 7, cardiac muscle, beta ) | chr14:23881947  -23904870 | NM_000257 | Familialhypertrophic cardiomyopathy,  myosin storage myopathy,  dilated cardiomyopathy | No CpGI |
| 35 | MTHFR(methylenetetra  hydrofolatereductase) | chr1:11845787  -11866160 | NM_005957 | Heart Defects Congenital,  Cardiovascular Diseases | CpGI |
| 36 | NF1(Neurofibromatosis 1) | chr17:29421945  -29549782 | ENSG  00000196712 | EC cushion defect, DORV | CpGI |
| 37 | NFATC1(Nuclear factor of  activated T cells,  cytoplasmic 1) | chr18:77160326  -77289323 | NM_172389 | VSD, valve defects | CpGI |
| 38 | NKX2-5(Csx/NK2 transcription  factor related, locus 5)* | chr5:172659107  -172662315 | NM_001166175 | ASD,* VSD, TOF, EP | CpGI |
| 39 | NR2F2 (COUP-TFII/nuclear  receptor subfamily 2,  group F, member 2) | chr15:96874111  -96883492 | NM_021005 | Atrial dysgenesis, ASD,  venous malformation  (cardinal vein obstruction) | CpGI |
| 40 | NRG1(Neuregulin 1) | chr8:32405728  -32622558 | NM_001160004 | Noncompaction,  dysmorphic trabeculae | CpGI |
| 41 | NRP1(Neuropilin-1) | chr10:33466419  -33623833 | NM_003873 | TGA, PTA | CpGI |
| 42 | NTF3(Neurotrophin 3) | chr12:5603298  -5604465 | NM_002527 | PTA, IAA, CT defects | CpGI |
| 43 | NFATC2(Nuclear factor  of activated T cells,  cytoplasmic 1) | chr20:50007765  -50179168 | NM_001136021 | Hypertrophy,immuno  deficiency,  Graft-Versus-Host Disease | CpGI |
| 44 | PAX3(Paired box gene 3) | chr2:223064606  -223163715 | NM_181459 | PTA, CT defects | CpGI |
| 45 | PCAF(p300/CBP-associated  factor) | chr3:20056528  -20169038 | U57317 | PTA, CT defects | CpGI |
| 46 | PDGFRA(Platelet-derived  growth factor receptor,  αpolypeptide) | chr4:55010806  -55079954 | NM_006206 | PTA, DORV,  VSD, noncompaction | CpGI |
| 47 | PITX2(Paired-like  homeodomain  transcription factor 2) | chr4:111997400  -112003074 | NM_000325 | PTA, DORV,  VSD, noncompaction | CpGI |
| 48 | JMJD6(jumonji domain containing 6) | chr17:75306037  -75320004 | NM_00  1081461 | DORV，VSD，PA : hypo，Thymus : hypo | CpGI |
| 49 | RARA(Rarα,retinoic  acid receptor,α) | chr17:38498271  -38513895 | NM_001024809 | IAA, VSD, PTA, DORV | CpGI |
| 50 | RARB(Rarβ ,retinoic acid  receptor,β) | chr3:25469754  -25639422 | NM_016152 | CT defects, VSD | No CpGI |
| 51 | RARG(Retinoic  acid receptor, gamma) | chr12:53604350  -53614197 | NM_001042728 | CT defects, VSD | CpGI |
| 52 | RXRA(Retinoid X  receptor,α) | chr9:137218316  -137332431 | NM_002957 | ASD, VSD,  AVSD, noncompaction,  PTA, AP window | CpGI |
| 53 | SEMA3C(Semaphoring  3C/sema domain,  immunoglobulin  domain, short basic  domain, secreted) | chr7:80371854  -80548667 | NM_006379 | IAA, PTA | CpGI |
| 54 | SLC2A10(Solute carrier  family 2, facilitated glucose  transporter member 10) | chr20:45338279  -45364985 | NM_030777 | Pulmonary Stenosis | CpGI |
| 55 | SMAD6(Smad6/mothers  against decapentaplegic  homologue 6) | chr15:66994674  -67074337 | NM_005585 | CT septation defects | CpGI |
| 56 | SOX11(SRY (sex determining  region Y)-box 11) | chr2:5832799  -5841517 | NM_003108 | DORV,VSD,Spectrum of  outflow tract,malformations | CpGI |
| 57 | SOX4(SRY (sex determining  region Y)-box 4) | chr6:21593972  -21598849 | NM_003107 | AVSD, TGA, semilunar  valve defects, PTA | CpGI |
| 58 | TBX1(T-box 1) | chr22:19744226  -19767068 | NM_080646 | Heart Defects  Congenital,DiGeorge Syndrome* | CpGI |
| 59 | TBX5(T-box 5) | chr12:114791735  -114846247 | NM_080717 | HOS*, ASD, VSD, TOF, EP | CpGI |
| 60 | TBX10(T-box 10) | chr11:67398774  -67407031 | NM_005995 | ASD, VSD, PFO | No CpGI |
| 61 | TBX20(T-box 20) | chr7:35271112  -35293711 | NM_001166220 | VSD*, Xenopus laevis | CpGI |
| 62 | TEAD1(Tef-1/TEA  domain family member 1) | chr11:12695969  -12966284 | NM_021961 | Noncompaction,  trabecular abnormality | CpGI |
| 63 | TEK(Tie2/endothelial-specific  receptor tyrosine kinase)* | chr9:27109147  -27230172 | NM_000459 | Venous malformations* | No CpGI |
| 64 | TGFB2(Transforming  growth factor, β2) | chr1:218518676  -218617961 | NM_003238 | VSD, DORV | CpGI |
| 65 | TGFBR3(Transforming  growth factor,  beta receptor III) | chr1:92145900  -92351836 | NM_003243 | Decreased cushion  mesenchyme transformation | CpGI |
| 66 | TLL1(Tolloid-like-1 ) | chr4:166794410  -166952111 | NM_001204760 | ASD* | CpGI |
| 67 | VCAM1(Vascular  cell adhesion molecule 1) | chr1:101185196  -101204601 | NM_080682 | Noncompaction, VSD | No CpGI |
| 68 | ZFPM2(FOG2  [friend of GATA] 2/zinc  finger protein, multitype 2) | chr8:106331147  -106816767 | NM_012082 | TA, ASD, VSD, PS,  TOF, AVSDTGA, | CpGI |
| 69 | ZIC3(Zic family member 3 ) | chrX:136648346  -136654259 | NM_003413 | DORV, ASD, AVSD | CpGI |
| 70 | FKBP1A(FK506  binding protein 1A, 12kDa) | chr20:1349621  -1373816 | NM_001199786 | HEART FAILURE,  Neurological Disorders | CpGI |
| 71 | PSEN1(Presenilin 1) | chr14:73603143  -73690399 | NM_007318 | Down Syndrome,  congenital malformation | CpGI |

**Additional file 1: Table S2. Primer sequences, position, product length, and CpG site used for MassArray quantitative methylation analysis**

|  | Gene | Forward primer(5′ →3 ′)^1^ | Reverse primer(5′ →3 ′)^2^ | Distance^3^ | Product  Length(bp) | No of CpG's | Coverage |
| --- | --- | --- | --- | --- | --- | --- | --- |
| 1 | ACVR1 | GTGTGTGTGTGTTGTGAATTTTTTT | CCAAAAACCCACATCTCTTCTTAAT | -388~20 | 409 | 11 | 10 |
| 2 | ACVR1B_M1 | ATTTTTAATTGTAGGGTTTTGGAGG | AACCCAAACTCCTAACACATACTACC | -709~-253 | 457 | 12 | 12 |
|  | ACVR1B_M2 | ATTGTATAGGTGGGGAATTTAAGGT | AACCCCAAAAAAACAACAACAC | -357~-68 | 290 | 21 | 16 |
| 3 | ANGPT1 | TTAAAAGTGTATTAAGGTGGATTGTTTT | CTATTTTCTCCCAAACCTCAATAAA | -146~139 | 286 | 6 | 5 |
| 4 | ASD1_M1 | AGTTTAGTTTTGTTGATTTTGTGGG | ACTCCCACCATAAACTCTTATCTCC | -692~-222 | 471 | 11 | 9 |
|  | ASD1_M2 | TAGATTATGTTGGGTAGTGGGAGAA | ACCCAATCCCAAATCTAAAAATAAA | -296~-134 | 431 | 26 | 22 |
| 5 | BCOR | AAGTTAAAAGTTGTTTTTTGTTAAGGA | AACCAAAACTATCAAACCCCAAC | -1601~-1109 | 493 | 37 | 32 |
| 6 | BMP4_M1 | GGAAATTTTTTAATAGTTTATGGAAGGTT | CTCCCTTTCTAAAAATAAAAACCCC | -519~-104 | 416 | 20 | 17 |
|  | BMP4_M2 | GGGGTTTTTATTTTTAGAAAGGGAG | CTCACCAAATAACCTTACTCACCAT | -128~284 | 413 | 25 | 23 |
| 7 | CFC1B_M1 | TTTTTTTTAGTGTTGATAAAAGGGGT | AAACACAAACAACAACCTAAAATCC | -709~-226 | 484 | 12 | 11 |
|  | CFC1B_M2 | TTTGTAGAGTAAGGGGGTTTTTTTT | TATAAATATTCCCTTCCCTTCCTCA | -330~155 | 486 | 8 | 8 |
| 8 | CITED2 | TGTAATAATAGGATGAGGAGGGTTG | CCATTTCCAATCCTAAAAATAAAAAA | -357~4 | 362 | 22 | 18 |
| 9 | COX6A2_M1 | ATTTTAGTATTGTGGGAGGTTGAGG | TCCCTTTCCTAAAACCTAAAATCAC | -406~48 | 455 | 5 | 5 |
|  | COX6A2_M2 | TAATAGGTGATTGGTTTAGAGAGGG | AAATAAACAAAATAAAAAAACCCCC | -4~206 | 211 | 10 | 6 |
| 10 | CRELD1 | GAGGTTTTGAATTTGATTTTTTTT | ACCATAAAACCATCTTTACCCAAAC | 149~630 | 482 | 15 | 10 |
| 11 | DVL2_M1 | GTTTGGTTAAGTGAGGAAGGAAAGT | AAATCCTCTAAACACCCAAACTAAA | -614~-143 | 472 | 14 | 14 |
|  | DVL2_M2 | AGTTTAGTTTGGGTGTTTAGAGGAT | CACCCAAAAAACTAATAACCCCTAC | -169~254 | 424 | 45 | 22 |
| 12 | ECE1_M1 | GTTGTTTTTATTTAGTTGGGTGGTG | TAATCTAATTCCCACAAATTCAACC | -368~91 | 460 | 8 | 4 |
|  | ECE1_M2 | TTGATTTTTTGATGTTTGGGAGTAG | ATAAATCAAACCCACCATACAACAC | -1~396 | 398 | 11 | 10 |
| 13 | EDN1_M1 | GGGGTTTTTTATTAGTGATAGGGAA | CCCATTAAAAACCTTTACTAAACCTA | -697~-227 | 471 | 12 | 8 |
|  | EDN1_M2 | TAGGTTTAGTAAAGGTTTTTAATGGG | AAAAAACAACTTCAAATCCCTCAA | -252~211 | 464 | 27 | 23 |
| 14 | EGFR_M1 | TTAGGGTTGTTGAATTAGGTTTGAA | CATCTAAAATAAAAACACCCAACTCC | -1045~-627 | 419 | 7 | 7 |
|  | EGFR_M2 | GGTGTTTGATAAGATTTGAAGGATTT | TTCCCAACACTACCCCTCTAAAC | -618~-258 | 361 | 20 | 20 |
| 15 | ELN_M1 | GGGTTGTGATTTTGATTTATGTAGAA | AACACCCCCAAATCTAACTAAAAAC | -557~-146 | 412 | 5 | 5 |
|  | ELN_M2 | TTTTGGGTAGAATTTGTTTTTAGTT | AAAACCTCTACCAAAACAACCCTC | -185~46 | 232 | 10 | 8 |
| 16 | EDNRA | TTTAAGGTAGGTTTTTTTTGAGGAGA | AACAACTAAACACAAAACCAAAACC | -436~-5 | 432 | 13 | 9 |
| 17 | ERBB2_M1 | GGATTTTGGTTTGATTTTTGTGTTA | AAAAACTTCATACTCCTAAACTCCTCC | -572~-73 | 500 | 28 | 24 |
|  | ERBB2_M2 | GGGGTGGGGTTTATTTGTTAGAT | AACCCTACCTCAAAAAAAATCATAAA | -319~147 | 467 | 32 | 26 |
| 18 | EVC_M1 | TTTATTTTTGGTTTTAGTGGGGATT | CTACCCCTACACTAAACATACCCAA | -546~-48 | 499 | 17 | 17 |
|  | EVC_M2 | TTTTAGTAAAGTTAAAGGGGTGGAAAT | CAAACTTTCTAACCTATACAATCTCCC | -145~71 | 217 | 11 | 8 |
| 19 | EVC2_M1 | TAGGATTTAGGTAGGGATGAGGAAG | AAATCCTAAAACACCCAAAAAATTC | -773~-386 | 388 | 12 | 11 |
|  | EVC2_M2 | GATGGGGAAATTTTGGATTTATATT | CCAAAACCACTACCAAAAAACC | -344~124 | 469 | 42 | 29 |
| 20 | FGF8 | GGTTGTTGTATTTGTTGGTTTTTTG | ACTACCTCTCTATACCTCAACTCCCTC | 124 ~335 | 212 | 18 | 13 |
| 21 | FOXC1 | TAGTTTTAATTTTTTGGGAGTGGTG | TTCTACCAACCCTACTTATTATCCC | 24~371 | 348 | 35 | 26 |
| 22 | GATA4 | GAGAGTTGAGTTTAAGAGGTTATTTTTTT | CCCTACCTACTAAACCTAAAAATTCC | -392~107 | 500 | 42 | 38 |
| 23 | GJA1 | TTTGTGATTTTAGTATTTTGGGAGG | CCTCAATCTTAAAACAAACAACTCC | -1529~-1055 | 475 | 8 | 8 |
| 24 | GJA5 | GAGGGATTTTTTGGGTAGTGTTAAT | ACCTATCAATTATTCCTCTTCCACC | -402~36 | 439 | 8 | 6 |
| 25 | HAND1_M1 | GAGGAGATTTGTTGGTTAGATGTTT | AATAAAAATTCCAACAATTCCCAAT | -887~-414 | 474 | 30 | 30 |
|  | HAND1_M2 | TGGGAATTGTTGGAATTTTTATTTA | ACCCAAAAACCTATTTAACCCTTCTA | -436 ~ 62 | 499 | 22 | 20 |
|  | HAND1_M3 | TAGAAGGGTTAAATAGGTTTTTGGGT | TCAACAACCAACTCTAAAAATAAAACC | 37~398 | 362 | 27 | 22 |
| 26 | HAS2_M1 | GAAATTTTTTTATGAGAAAGGTTTTGA | AAAAAACCACCACCTAATTCCTTAC | -693~-313 | 381 | 16 | 14 |
|  | HAS2_M2 | AAAGTAAGGAATTAGGTGGTGGTTT | ATAAAAAACTCAACAAAACCCAAAA | -340~132 | 473 | 16 | 11 |
| 27 | HEY2_M1 | TTTAATTAAGGGATAGGTAAATGGAA | CCTAAAACAAAACCTAAAATCATCA | -569~-87 | 483 | 9 | 9 |
|  | HEY2_M2 | TGATGATTTTAGGTTTTGTTTTAGG | ATAAACCCCAACTCCCCTATC | -111~258 | 370 | 39 | 36 |
| 28 | HOXA3 | AGGTTTTAGGTTGGAGTTGAGATGT | CCCCTTCAAAACTCTTAAAACAATC | -1055~-609 | 447 | 11 | 11 |
| 29 | HSPG2_M1 | ATTTTATTATTTTGGGAGGGTAAGG | CAAAAACACAAAAAACAACCCTC | -1084~-651 | 434 | 15 | 14 |
|  | HSPG2_M2 | TGTTTTTTGTGTTTTTGGTGGAG | CTAAACCTTTATCTAAACCCCCAAC | -667~-169 | 499 | 48 | 37 |
| 30 | JAG1_M1 | GGTAAGTTTGGTTTTAGGAAAGTTTTT | AAACCAAAACCCCACACCAAC | -679~-229 | 451 | 53 | 41 |
|  | JAG1_M2 | TGATTAGGATGGTTTTAATTTTTTGA | TAAAATTTCCATCAACACCAATCTC | -1987~-1788 | 200 | 8 | 8 |
| 31 | MED13L_M1 | TGTTGTTTATGAGTTTTGTTTTAGTAGT | TCCAAACATAAAACAATCAACTATTTC | -1828~-1360 | 469 | 10 | 10 |
|  | MED13L_M2 | GATTTATTTTTTGAGGATTGGGAGA | CCAAACTCTTAAACAAAATTTCACA | -1316~-951 | 366 | 8 | 8 |
| 32 | MEF2C | TGTTTTGTTTTAAGATTATTTTTGG | AAAACTTTATTATCCAAACCCTAACAA | -358~119 | 478 | 8 | 7 |
| 33 | MYH6_M1 | ATTAGGAGTGGGGTGTAGGTTAGTT | ACAAAACAAACCAAAAAATCAAAAA | -565~-117 | 449 | 6 | 5 |
|  | MYH6_M2 | TTTTTGATTTTTTGGTTTGTTTTGT | AAAACACCTACTATTACACCCTCCC | -141~267 | 409 | 7 | 5 |
| 34 | MYH7 | GGGGAGTAGTGTTTAGGGTTAGAAG | TTATCCCAAAATAAAACCTCCAACT | -1783~-1320 | 464 | 8 | 6 |
| 35 | MTHFR_M1 | GATTGAGATTAGGAGTGGTTGTAGA | AACAAAAAACCAAAATCAATCTTC | -739~-240 | 500 | 45 | 36 |
|  | MTHFR_M2 | AAGATTGATTTTGGTTTTTTGTTTG | AAAATATCCCCAAAATTTAACACAC | -262~167 | 430 | 28 | 25 |
| 36 | NF1_M1 | ATTTTAGTATTTTGGGAGGTTGAGG | AAATCTCCTAAAACAAAATCCTTCC | -1338~-918 | 421 | 11 | 11 |
|  | NF1_M2 | AGATTTGGTGGTTAGATTTTGATTG | CCCTAAAACTAACCTTTCTCCCAAC | -557~-308 | 250 | 10 | 8 |
| 37 | NFATC1_M1 | AGTTAGGTGTGGTGATATGTGTTTG | CTCAAATCACCAATAAATCAATCAA | -1599~-1179 | 421 | 8 | 8 |
|  | NFATC1_M2 | TTTTAAAGATTTTTGATGGGAAAAG | AAAATACCAAATCCCTTCTCCTAAA | -688~-194 | 495 | 31 | 27 |
| 38 | NKX2-5 | AGGAGGGTTTTGGATTTTTTTT | ATTTATTCCCAAACCTCTACTCCTC | -40~426 | 467 | 29 | 28 |
| 39 | NR2F2 | AATTGTTTATAAATTTGGTTGGTTT | ATCAATATACACAAACAAAAACTTCAC | -1574~-1090 | 485 | 5 | 5 |
| 40 | NRG1 | TTTTATTAGAGGGAGGATAAGGGAA | TTCCTTCCAAATACACAAAAAAAAC | -976~-527 | 450 | 13 | 13 |
| 41 | NRP1_M1 | GAGTTTAGGAAATTGGGTTTTGTTT | AAACTCCTTAATTTCCACTCACAATA | -1544~-1141 | 404 | 8 | 8 |
|  | NRP1_M2 | TAGTTGAGAAGAAGGAGGAAATTGGT | CACACACAACCAAAATACAACCTAA | -286~100 | 387 | 26 | 24 |
| 42 | NTF3_M1 | TGATATTGTAATATGGGGGTAATTGA | AACAAAACAACTCCCCTCTAAACAT | -474~-74 | 401 | 10 | 10 |
|  | NTF3_M2 | TTTTTTTAGAATGTTTAGAGGGGAG | AAAAACCTCAACTTTAAACAAAATACTCT | -108~277 | 386 | 6 | 6 |
| 43 | NFATC2_M1 | TTAATTTTTTTTGGTATATGGGGAA | ACTACAATCCAAACCCACTAAAACA | -1715~-1221 | 495 | 16 | 13 |
|  | NFATC2_M2 | GAGGTTTTTTTTGATTATTTAAGGGAT | TTTTACCCTAAAAAACCCACTAACC | -735~-289 | 447 | 11 | 9 |
| 44 | PAX3_M1 | AGAGAGAGAGGAGTGGAGAATTTTT | TAAAAACTAAAATATCCCCAACAAA | -1389~-1015 | 375 | 13 | 13 |
|  | PAX3_M2 | TTTGGGGTTGTTTTTTTTAGTTTTT | CCTAAATCTCCTCCTATAATAACACC | -267~15 | 283 | 19 | 19 |
| 45 | PCAF | AGTAGGGTATTTTTGAAGGGTTTTG | TCTTCCCATAACAATTTCTAAATCAA | -913--428 | 486 | 11 | 8 |
| 46 | PDGFRA_M1 | GGGTATATATGGTGGTTTGGTTTTT | AAAATCAACATCTACCTCCTCTACAAA | -2421~-1961 | 461 | 29 | 29 |
|  | PDGFRA_M2 | TTAGATAAGTGATTTTAAGGGAGTAAAGG | CAAAATAAATTTACCTCCCCATTTT | -590~-156 | 435 | 10 | 10 |
| 47 | PITX2_M1 | TTTTTGAAATTGGTTATTTGGTAAT | CAAAAAAAACAAAACCCTAAAAAAA | -1034~-546 | 489 | 11 | 10 |
|  | PITX2_M2 | GGGAGTTTGTTTGTGTAGATTTAGTG | CAACCCTAACAAAAAAATATCAAAA | -230~241 | 472 | 43 | 36 |
| 48 | JMJD6_M1 | AAAGGAAAGAGGTTTTTATTTTTATAG | AACTACAAACATTAACCACCACACC | -1995~-1571 | 425 | 11 | 9 |
|  | JMJD6_M2 | TAAGAAGGGGAAAGTTTAGATTTGG | TTCCATTAACTTTAACTCCCCTAAAA | -162~96 | 259 | 26 | 20 |
| 49 | RARA | TTTTAAGGAAGGAAGATAGGTAGGA | TATCCCTAACTTTAACCCAAAAACC | -1688~-1221 | 468 | 28 | 23 |
| 50 | RARB | GAAATTATGTTGGGAAGAAGTAAGG | AAAAAAAACAAATTCCAACAAAACT | -168~206 | 375 | 12 | 10 |
| 51 | RARG | TGAGTTGTGGAGAGTATGTTTGTAA | AAAAAAAATCCCCAACAAAAAAAA | -308~109 | 418 | 23 | 17 |
| 52 | RXRA | TAGTAGTATATGGTGGGAAGGTTGG | CCAAAAAAACCCTCTATTCCTAAAA | -1014 ~-615 | 400 | 15 | 12 |
| 53 | SEMA3C_M1 | GATTTATTTTGAGGTGTGGTTAATTG | AAATAAAATCCAAATTATATACTCTCCAC | -1974~-1486 | 489 | 7 | 7 |
|  | SEMA3C_M2 | TTATTTAGGAGGTTGAGGTAGGAGAA | TCACTACAACCTTTTAAAACAAAAAAA | -367~-138 | 230 | 7 | 6 |
| 54 | SLC2A10_M1 | ATTTTTAGGTTAGGTTGAGGGAAGA | AAAAAATCTAATTACACTAAAACCCTCC | -1933~-1530 | 404 | 7 | 7 |
|  | SLC2A10_M2 | GGGTTTTTGTTTTTGTTGTTTTTTT | TACCCCAATCACTAATCCACACTTA | -615~-154 | 462 | 13 | 10 |
| 55 | SMAD6_M1 | TGATTGAAAGGGGTTATAAGATAGG | CATTTACAAAAAAAATTCAAAACCC | -1849~-1464 | 386 | 15 | 14 |
|  | SMAD6_M2 | TTTAGATTGGTATATGATGGGAGGT | CAAAACCTAAATCCCTAAATTTACATACA | -106 ~ 352 | 459 | 35 | 29 |
| 56 | SOX11 | TGGTTTTTATGGTTTTGGAAAGAAT | CCCCCAACACTAAATATCTAAAAAC | -2102~-1726 | 377 | 10 | 10 |
| 57 | SOX4 | AAATTAATGGAATGGTAGGGTTGTT | CCTATAATCCCAACACTTTAAAAAA | -1776~-1434 | 343 | 5 | 5 |
| 58 | TBX1_M1 | TTTTGAGGTTTAGAGGAGGAGTTTT | CCTACCCCAAAAAAATCCAATATAC | -853~-365 | 489 | 18 | 13 |
|  | TBX1_M2 | TTTTAGTATATTGGATTTTTTTGGGG | AATATTCCTCCCTCCCTCACCTAAC | -394 ~ 63 | 458 | 28 | 20 |
| 59 | TBX5_M1 | GGTTGTTTTTTTATTTTATTGGGGT | AACCCAACCAAATTTACTTAACTCAT | -1968~-1479 | 490 | 10 | 9 |
|  | TBX5_M2 | TATTTTAGAGAGGGAAAGGGTAAGG | AACTACAAAAACTCCTACACAAATCA | -763~-277 | 487 | 22 | 20 |
| 60 | TBX10 | TTAGGTTTGGATTTTTGTAGATGTTT | CTCAACTCAACCCTCAAATACTCAC | -415~-11 | 405 | 17 | 15 |
| 61 | TBX20_M1 | TTTGAGTGTGTATGTTAGTTTGAGTTT | CTCCTATTTTCCCTAAAAAAACCCT | -945~-635 | 311 | 23 | 22 |
|  | TBX20_M2 | GTGAGATTAGGTGGGGATGTTTAT | TCCTTCCTCCCTCTAAAACTAAAAA | -214~167 | 382 | 42 | 32 |
| 62 | TEAD1 | TTGGAGAAGATTTTGTTAGTAGGGT | AAACCTAAATTTCTTCCAAAAAACAA | -728~-343 | 386 | 27 | 24 |
| 63 | TEK | ATAATTTTGGATTTTGGTTTTTTGG | TAAAAACACTCAAACCCTATCCATC | -280~174 | 455 | 8 | 7 |
| 64 | TGFB2 | TGGGAGGTTGTGATTGAGTTATATT | TTTCTCTTATCAAAAACTTCTAAAACTCC | -1202~-833 | 370 | 15 | 14 |
| 65 | TGFBR3_M1 | TTTAGGAGTTTGAGATTAGTTTGGGT | AAATTTCAAAAACCAAACTTCCAAT | -1265~-861 | 405 | 10 | 9 |
|  | TGFBR3_M2 | GAGTGGTGTTTTGGTGTTAGTAGTT | AAACTTTCCAAAACTCCCTAAAAAA | -761~-307 | 455 | 19 | 19 |
| 66 | TLL1_M1 | GTTTGTTGGGGGAGAAGTTATTTTA | AAAAACTAAAATCCTCAAAACTACAA | -961~-521 | 441 | 12 | 12 |
|  | TLL1_M2 | GTAAAATTTTGTTGGTTAGGAGATT | ATTCATACAATCAAAATCAAAATCC | -519~-117 | 403 | 12 | 11 |
| 67 | VCAM1 | TTTTTTTAGTAAAGATAGTTTTTTGGAG | TCAACTCCTAAAACCAATAAAACCC | -263~32 | 296 | 5 | 5 |
| 68 | ZFPM2 | GGGATTTATGTGAATTGTAGTGGAG | AAAATTCTATAATCCCACCCTACCC | -1752~-1323 | 430 | 6 | 6 |
| 69 | ZIC3_M1 | TTTTTTAATTTGGGGAGAGAGTTTT | ATCACATCCCTAACTAAACCAAACC | -1811~-1489 | 323 | 8 | 7 |
|  | ZIC3_M2 | GGATATGTTTTTAAGGTGGTGAGGT | AAACTAATAAAATCAATCACTCACTCCTC | -470~-7 | 464 | 53 | 35 |
| 70 | FKBP1A | TTTTTTGGGTAGGGAGATGTTTAAT | AAAAATAATTTCCACCTACACTCCC | -166~191 | 358 | 43 | 24 |
| 71 | PSEN1_M1 | TTGTTGTTTAGGTTGGAATGTAATG | TTCCAACACTTTAAAAAACCAAAAT | -1884~-1647 | 238 | 7 | 7 |
|  | PSEN1_M2 | TGTTTTGTTTTTTAGGTTGGAGTGT | TCATAAAATTAAAAATTCTAAACCAACC | -1031~-839 | 193 | 7 | 7 |
|  | PSEN1_M3 | TTGGGTTTAATTTATATAGGGGTTTT | TAACTCAAATTCCTTCCAAACCAAC | -229~100 | 330 | 26 | 21 |

^1^10-mer tag : cagtaatacgactcactatagggagaagg ; ^2^T7 promoter tag: aggaagagag were added.^3^Relative to transcription start site.

**Additional file 1: Table S3**. Primer sequences and product length for QPT-PCR analysis

| Genes | Forward primer(5′ →3 ′) | Reverse primer (5′ →3 ′) | Product length(bp) |
| --- | --- | --- | --- |
| *EGFR* | CGTGCCCTGATGGATGAAGA | CGGTGGAATTGTTGCTGGTTG | 145 |
| *EVC2* | TCACGGTCCAAGAGTAAAAGCA | TTTCCACCAGGTCTTCAGAGGC | 100 |
| *NFATC2* | GGTTCCTACCCCACAGTCATTC | TCTGGTCCAAGTTCTGCTCCTG | 136 |
| *NR2F2* | CCCCTCAACTGCCACTCGTA | GCCAGTTCGCAAATGTTCTC | 137 |
| *TBX5* | ACAGTCCTTTCAGCAGCGAGTC | AGTGGGTATGGGTTGGGTGG | 127 |
| *CFC1B* | TGTCAGGCTTCTGTTTACGG | GTGGCAACCTTGGTGACTTC | 111 |
| *GJA5* | GGGAGGAAGGGAATGGAAGGA | ATGCAGGGTGGTCAGGAAGA | 143 |
| *B2M* | TGCTGTCTCCATGTTTGATGTATCT | TCTCTGCTCCCCACCTCTAAGT | 161 |
| *GAPDH* | AGAAGGCTGGGGCTCATTTG | AGGGGCCATCCACAGTCTTC | 220 |

**Additional file 1: Table S4. Mean and median methylation levels for 113 amplicons (71 candidate genes) in 10 TOF cases and 6 Controls**

**P* < 0.05, ***P* < 0.01, ****P* < 0.001 (Mann-Whitney test)；IQR，interquartile range.

| **Gene amplicon** | **Genomic**  **Location** | **Accession**  **numbers** | **Control: Mean ± SD ;**  **median(IQR);N** | **TOF: Mean ± SD;**  **median(IQR),N** | ***P* value** | **Position** |
| --- | --- | --- | --- | --- | --- | --- |
| ACVR1 | chr2:158592958  -158731623 | NM_  001111067 | 6.23 ±2.18;  5.67(5.09-7.38); N=6 | 7.46 ± 2.91; 6.75(5.48-8.42); N=10 | 0.3279 | CpGI |
| ACVR2B_M1 | chr3:38495790  -38534633 | NM_  001106 | 18.88±2.96;  19.72(16.92-20.88);N=6 | 20.11 ± 6.91; 19.41(16.32-24.05); N=10 | 0.9578 | CpGI |
| ACVR2B_M2 |  |  | 7.65 ±1.93;  7.50(5.80-9.28); N=6 | 7.71 ±2.42;  7.20(5.73-8.73); N=10 | 0.7441 | CpGI |
| ANGPT1 | chr8:108261721  -108510283 | NM_  001199859 | 54.60 ±19.11; 63.33(38.50-66.33);N=5 | 64.04 ± 6.99; 62.17(58.00-70.33);N=8 | 0.4531 | TSS |
| ASD1_M1 | chr5 | GC06  U990006 | 84.64 ± 13.20; 88.75(73.19-94.03);N=5 | 92.01 ± 3.68; 93.85(89.66-94.63); N=10 | 0.3097 | CpGI shore |
| ASD1_M2 |  |  | 26.41 ± 11.05; 25.36(18.46-34.89);N=5 | 21.15 ±6.13;  22.20(17.84-24.68); N=10 | 0.2544 | CpGI |
| BCOR | chrX:39910499  -39956656 | NM_  017745 | 5.20 ± 2.08; 5.56(3.24-6.99);N=5 | 6.92 ±2.07; 6.78(5.42-7.51); N=9 | 0.2977 | CpGI |
| BMP4_M1 | chr14:54416455  -54423554 | NM_  001202 | 15.63 ± 4.51; 15.45(11.80-19.75);N=6 | 14.39 ± 5.47; 15.00(10.32-19.04); N=8 | 0.8518 | CpGI |
| BMP4_M2 |  |  | 10.78 ± 1.95;  11.06(9.03-12.38); N=5 | 12.83 ± 3.39; 11.19(10.64-15.23); N=10 | 0.5135 | CpGI |
| CFC1B_M1 | chr2:131350353  -131357251 | NM_  001079530 | 81.17 ± 7.99; 83.56(74.56-86.97);N=6 | 79.10 ± 8.17; 80.06(73.75-83.89); N=10 | 0.4278 | TSS |
| CFC1B_M2 |  |  | 48.89±11.02; 48.16(40.36-59.25);N=6 | 69.40±15.58; 67.71(54.67-80.67); N=10 | 0.0375* | TSS |
| CITED2 | chr6:139693397  -139695785 | NM_  006079 | 4.13 ± 1.36; 4.07(2.95-5.04); N=6 | 4.13±1.21; 3.55(3.49-4.62); N=10 | 0.9578 | CpGI |
| COX6A2_M1 | chr16:31439052  -31439749 | NM_  005205 | 41.79 ±14.14; 41.38(26.88-57.50);N=6 | 40.42 ± 14.14; 36.13(31.56-55.69); N=10 | 0.7925 | CpGI shore |
| COX6A2_M2 |  |  | 26.83 ±16.76; 23.00(13.75-38.63);N=6 | 23.94 ±9.97;  22.25(20.75-32.63); N=9 | 0.8639 | CpGI |
| CRELDd1 | chr3:9975524  -9987097 | NM_  015513 | 18.58 ± 9.62; 14.17(10.97-30.50);N=6 | 20.48 ± 11.57; 20.92(8.06-31.14); N=8 | 0.8972 | TSS |
| DVL2_M1 | chr17:7128661  -7137863 | NM_  004422 | 25.26 ± 3.09; 24.95(22.42-28.44);N=6 | 22.73 ±4.29;  22.62(18.91-26.52); N=10 | 0.3132 | CpGI |
| DVL2_M2 |  |  | 9.05 ±2.91; 9.09(6.81-11.22); N=6 | 13.01 ± 2.55; 12.44(11.45-14.30);N=9 | 0.0176* | CpGI |
| ECE1_M1 | chr1:21545077  -21606042 | NM_  001397 | 59.64 ± 30.44; 64.33(43.83-80.38);N=6 | 61.63 ±24.38; 72.33(41.17-81.50); N=9 | 1 | TSS |
| ECE1_M2 |  |  | 68.76 ±17.78; 77.21(57.65-78.98);N=6 | 68.36 ±8.82;  66.13(60.61-75.18); N=10 | 0.6354 | TSS |
| EDN1_M1 | chr6:12290529  -12297427 | NM_  001168319 | 10.85 ± 2.84;  11.13(8.16-13.56); N=6 | 9.86 ± 3.33;  10.38(6.63-12.42); N=9 | 0.5553 | CpGI |
| EDN1_M2 |  |  | 11.23 ± 3.08;  11.46(8.29-14.06); N=5 | 7.52 ± 1.73;  7.29(6.38-8.96); N=9 | 0.0290* | CpGI |
| EGFR_M1 | chr7:55086714  -55270769 | NM_  201282 | 50.04 ± 8.13; 52.75(44.00-56.35);N=6 | 59.30 ±9.52;  59.17(55.57-64.92); N=10 | 0.040* | CpGI |
| EGFR_M2 |  |  | 15.67 ±4.39; 14.58(11.96-20.21);N=4 | 15.42 ± 4.17; 15.15(11.73-18.85); N=10 | 1 | CpGI |
| ELN_M1 | chr7:73442427  -73484236 | NM_  000501 | 43.00 ± 25.77; 48.10(17.88-63.73);N=6 | 62.41 ± 21.14; 56.25(50.25-85.40); N=7 | 0.2833 | CpGI shore |
| ELN_M2 |  |  | 31.92 ± 24.22;  31.19(5.57-58.64); N=6 | 42.26 ± 15.69; 48.07(34.07-53.98); N=10 | 0.5622 | CpGI shore |
| EDNRA | chr4:148402069  -148466106 | NM_  001957 | 38.70±9.95; 42.25(34.69-44.42);N=6 | 46.76±7.77;  46.13(40.63-50.88); N=9 | 0.049* | CpGI shore |
| ERBB2_M1 | chr17:37856254  -37884915 | NM_  004448 | 16.13 ± 4.86; 13.50(12.30-21.27);N=5 | 13.34 ±3.35;  12.83(10.47-16.71); N=4 | 0.4127 | CpGI |
| ERBB3_M2 |  |  | 5.83 ± 2.79;  5.50(3.75-7.25); N=6 | 3.70 ± 2.31;  4.00(1.75-5.00); N=10 | 0.1384 | CpGI |
| EVC_M1 | chr4:5712924  -5816031 | NM_  153717 | 15.40 ± 7.25; 14.27(10.88-19.78);N=6 | 12.96 ± 2.94; 11.77(10.58-16.10); N=9 | 0.4559 | CpGI |
| EVC_M2 |  |  | 4.61 ± 3.55;  2.92(2.42-7.21); N=6 | 3.85 ± 1.63;  3.58(2.71-4.54); N=10 | 0.8281 | CpGI |
| EVC2_M1 | chr4:5564146  -5711275 | NM_  001166136 | 26.81 ± 7.03; 29.63(18.84-32.25);N=6 | 39.63 ± 9.92; 36.38(31.88-48.81); N=9 | 0.0190* | CpGI |
| EVC2_M2 |  |  | 7.75 ± 2.38;  7.36(5.59-10.29); N=3 | 7.40 ± 2.74;  6.63(5.21-10.82); N=7 | 0.8333 | CpGI |
| FGF8 | chr10:103529887  -103535759 | NM_  033164 | 6.74 ± 2.48;  6.07(4.71-8.86); N=6 | 5.64 ± 0.82;  5.71(4.79-6.46); N=10 | 0.6642 | CpGI |
| FOXC1 | chr6:1610681  -1614129 | NM_  001453 | 8.86 ±1.92;  8.46(7.17-10.94); N=3 | 9.60 ±1.05;  9.89(8.72-10.41); N=7 | 0.5167 | CpGI |
| GATA4 | chr8:11561717  -11617509 | NM_  002052 | 6.91 ± 0.04;  6.91(6.88-6.94); N=2 | 4.84 ±1.39;  4.65(3.66-6.21); N=4 | 0.1333 | CpGI |
| GJA1 | chr6:121756745  -121770873 | NM_  000165 | 60.49 ± 5.24; 61.09(55.63-64.74);N=6 | 54.18 ± 15.14; 58.50(42.30-63.07); N=10 | 0.4278 | TSS |
| GJA5 | chr1:147228332  -147245484 | NM_  005266 | 32.56 ± 3.83; 32.83(29.00-36.08);N=6 | 48.40 ± 5.71; 48.33(44.25-54.00); N=10 | 0.0225* | TSS |
| HAND1_M1 | chr5:153854532  -153857824 | NM_  004821 | 14.64±9.45; 12.71(6.73-24.48); N=4 | 36.45± 17.43; 30.77(21.59-51.65); N=10 | 0.0360* | CpGI |
| HAND1_M2 |  |  | 9.80±1.31;  9.27(9.00-10.88); N=5 | 9.55±2.62;  9.56(8.30-11.42); N=10 | 0.9631 | CpGI |
| HAND1_M3 |  |  | 16.99±3.51; 16.77(13.75-20.47);N=4 | 12.57±4.38; 12.13(8.11-15.98); N=10 | 0.0797 | CpGI |
| HAS2_M1 | chr8:122625271  -122653630 | NM_  005328 | 7.65 ± 1.77;  7.55(6.59-8.75); N=6 | 7.16 ± 2.49;  6.77(5.36-9.56); N=10 | 0.6642 | CpGI |
| HAS2_M2 |  |  | 4.56 ± 1.76;  3.75(3.31-6.65); N=6 | 8.52 ± 3.21;  7.68(5.78-11.38); N=8 | 0.0200* | CpGI |
| HEY2_M1 | chr6:126070732  -126082415 | NM_  012259 | 7.12 ± 1.75;  7.14(5.25-8.64); N=6 | 6.54 ± 4.94; 5.79(2.46-9.50);N=10 | 0.447 | CpGI |
| HEY2_M2 |  |  | 5.79 ± 1.18;  5.56(5.05-6.36); N=6 | 6.44 ± 2.04;  5.95(4.70-7.66); N=9 | 0.5553 | CpGI |
| HOXA3 | chr7:27145809  -27159214 | NM_  030661 | 45.87 ± 7.51; 47.33(38.44-52.56);N=5 | 31.50 ± 6.97; 31.17(26.33-37.78); N=8 | 0.0190* | TSS |
| HSPG2_M1 | chr1:22148737  -22263750 | NM_  005529 | 76.52 ± 12.78; 75.70(63.64-88.99);N=6 | 71.65 ± 11.59; 76.20(65.97-80.68); N=9 | 0.6869 | CpGI |
| HSPG2_M2 |  |  | 4.56 ± 1.76;  3.75(3.31-6.65); N=6 | 8.52 ± 3.21;  7.68(5.78-11.38); N=8 | 0.0200* | CpGI |
| JAG1_M1 | chr20:10618332  -10654694 | NM_  000214 | 2.77 ± 1.52;  2.77(1.69-3.85); N=2 | 4.83 ± 2.67;  4.08(2.73-7.35); N=6 | 0.3144 | CpGI |
| JAG1_M2 |  |  | 93.24 ± 3.75; 93.83(89.83-96.53);N=6 | 92.25 ± 2.91; 93.42(89.21-94.42);N=10 | 0.5496 | CpGI |
| MED13L_M1 | chr12:116396381  -116714991 | NM_  015335 | 81.66 ± 5.77; 82.57(76.69-86.17);N=5 | 88.92 ± 4.85; 90.94(85.64-91.78); N=10 | 0.0400* | CpGI shore |
| MED13L_M2 |  |  | 49.73 ± 18.92; 52.33(34.00-64.17);N=5 | 48.27 ± 12.18; 49.17(41.15-56.34); N=10 | 0.5941 | CpGI |
| MEF2C | chr5:88014058  -88119744 | NM_  001193348 | 83.93 ± 8.21; 85.80(75.55-90.85);N=6 | 83.18 ± 5.87; 83.80(80.60-86.70); N=10 | 0.6354 | TSS |
| MYH6_M1 | chr14:23851199  -23877486 | NM_  002471 | 39.58±11.11; 39.00(30.63-46.25);N=6 | 22.75 ± 14.59; 18.25(7.38-36.25); N=10 | 0.0355* | TSS |
| MYH6_M2 |  |  | 56.42 ± 21.33; 56.42(41.33-71.50);N=2 | 63.07 ± 16.26; 53.75(52.75-69.75); N=7 | 0.8889 | TSS |
| MYH7 | chr14:23881947  -23904870 | NM_  000257 | 25.23±8.96; 27.28(16.00-32.88);N=6 | 27.55 ± 23.15; 24.25(15.02-29.63);N=10 | 0.6501 | TSS |
| MTHFR_M1 | chr1:11845787  -11866160 | NM_  005957 | 7.07 ± 1.87;  7.64(5.09-8.48); N=4 | 8.03 ± 2.03;  8.27(6.12-9.68); N=8 | 0.5697 | CpGI |
| MTHFR_M2 |  |  | 10.53 ±1.87;  9.70(9.42-12.46); N=4 | 8.16 ± 2.02;  8.40(6.02-10.07); N=8 | 0.2828 | CpGI |
| NF1_M1 | chr17:29421945  -29549782 | ENSG000  00196712 | 94.22 ± 1.20; 94.22(93.39-94.89);N=6 | 92.82 ± 3.15; 93.00(89.86-95.69);N=10 | 0.5622 | CpGI |
| NF1_M2 |  |  | 6.17 ± 3.41;  5.50(2.95-9.35); N=6 | 4.68 ±1.83;  4.40(3.20-6.35); N=10 | 0.5873 | CpGI |
| NFATC1_M1 | chr18:77160326  -77289323 | NM_  172389 | 85.27 ± 5.35; 84.79(80.43-90.59);N=4 | 91.86 ± 5.04; 93.00(88.25-95.75); N=9 | 0.0503* | CpGI shore |
| NFATC1_M2 |  |  | 20.17 ± 4.06; 19.09(17.25-23.46);N=5 | 21.11 ± 4.87; 22.14(15.32-25.95); N=7 | 0.7551 | CpGI |
| NKX2-5 | chr5:172659107  -172662315 | NM_  001166175 | 22.67 ± 2.28; 22.72(20.46-24.83);N=4 | 38.01 ± 12.45; 33.38(30.91-51.49); N=8 | 0.0081** | CpGI |
| NR2F2 | chr15:96874111  -96883492 | NM_  021005 | 60.20 ± 11.30; 62.50(54.35-68.30);N=6 | 45.40 ± 11.76; 45.00(34.30-53.80); N=10 | 0.0312* | CpGI shore |
| NRG1 | chr8:32405728  -32622558 | NM_  001160004 | 22.75 ± 6.55; 24.00(16.00-28.25);N=4 | 10.39 ± 4.72;  9.50(6.00-15.75); N=9 | 0.0196* | CpGI |
| NRP1_M1 | chr10:33466419  -33623833 | NM_  003873 | 19.29 ±15.66;  12.00(8.43-33.79); N=5 | 5.80 ± 3.07;  5.57(2.75-8.79); N=10 | 0.0127* | CpGI shore |
| NRP1_M2 |  |  | 9.03 ± 3.62;  8.57(5.80-12.73); N=4 | 10.30 ± 4.41; 10.19(6.17-14.41);N=10 | 0.6354 | CpGI |
| NTF3_M1 | chr12:5603298  -5604465 | NM_  002527 | 92.03 ± 2.25; 92.75(89.55-93.93);N=6 | 89.60 ± 2.47; 89.80(87.35-91.10); N=9 | 0.111 | CpGI shore |
| NTF3_M2 |  |  | 84.85 ± 6.24; 85.80(78.45-90.30);N=4 | 80.35 ±7.41;  81.90(72.68-87.00); N=6 | 0.3923 | CpGI |
| NFATC2_M1 | chr20:50007765  -50179168 | NM_  001136021 | 91.75 ± 4.72;  94.18(87.41-94.86); N=5 | 94.51 ± 2.75; 94.73(92.59-97.23); N=8 | 0.2844 | CpGI shore |
| NFATC2_M2 |  |  | 20.83 ± 6.76; 21.80(17.50-25.53);N=6 | 34.91 ± 14.90; 30.39(24.91-42.56); N=10 | 0.0252* | CpGI |
| PAX3_M1 | chr2:223064606  -223163715 | NM_  181459 | 9.09 ± 3.23;  9.56(6.17-12.07); N=6 | 10.18 ± 3.36;  9.87(7.94-12.00); N=10 | 0.7128 | CpGI |
| PAX3_M2 |  |  | 4.18 ± 2.66;  3.25(2.89-4.99); N=6 | 7.93 ± 6.75;  5.64(4.05-8.86); N=10 | 0.0392* | CpGI |
| PCAF | chr3:20056528  -20169038 | U57317 | 21.13 ± 26.01;  11.83(3.64-37.04); N=6 | 16.18 ± 9.13; 14.44(10.03-22.25); N=10 | 0.7128 | CpGI shore |
| PDGFRA_M1 | chr4:55010806  -55079954 | NM_  006206 | 8.41 ± 18.46;  8.28(6.63-10.32); N=3 | 12.91 ± 5.00;  12.83(9.11-16.17); N=9 | 0.1455 | CpGI |
| PDGFRA_M2 |  |  | 23.03 ± 9.54; 18.31(16.23-32.22);N=6 | 12.21 ± 5.67;  14.75(5.88-16.63); N=9 | 0.0337* | CpGI |
| PITX2_M1 | chr4:111997400  -112003074 | NM_  000325 | 16.97 ± 11.48;  15.56(9.22-22.78); N=6 | 16.58 ± 8.96; 14.88(11.22-23.00); N=10 | 0.7925 | CpGI shore |
| PITX2_M2 |  |  | 4.62 ± 1.53;  4.36(3.45-5.93); N=6 | 5.37 ± 1.77;  5.45(3.86-6.57); N=9 | 0.4559 | CpGI |
| JMJD6_M1 | chr17:75306037  -75320004 | NM_00  1081461 | 91.63 ±3.33; 92.63(90.25-93.50);N=6 | 89.09 ± 4.46; 90.06(87.53-91.78); N=10 | 0.0925 | CpGI shore |
| JMJD6_M2 |  |  | 3.12 ± 0.86;  3.33(2.24-3.80); N=4 | 6.34 ± 2.83; 6.50(3.51-9.04); N=9 | 0.0755 | CpGI |
| RARA | chr17:38498271  -38513895 | NM_00  1024809 | 10.69 ± 7.86;  7.86(5.24-16.28); N=6 | 11.59 ± 4.55;  12.06(6.99-15.16); N=9 | 0.5287 | CpGI |
| RARB | chr3:25469754  -25639422 | NM_  016152 | 77.40 ± 16.00; 81.21(58.57-91.21);N=6 | 87.24 ± 5.78; 85.93(83.64-91.68); N=10 | 0.3286 | TSS |
| RARG | chr12:53604350  -53614197 | NM_00  1042728 | 4.28 ± 1.64;  3.78(2.85-6.18); N=6 | 3.79 ±2.04;  2.95(2.46-4.84); N=10 | 0.3676 | CpGI |
| RXRA | chr9:137218316  -137332431 | NM_00  2957 | 59.88 ± 4.57; 60.05(54.93-64.48);N=6 | 61.56 ± 4.81; 63.30(58.80-64.80); N=9 | 0.4559 | CpGI shore |
| SEMA3C_M1 | chr7:80371854  -80548667 | NM_00  6379 | 88.80 ± 7.76; 86.00(82.30-97.90);N=6 | 90.96 ± 6.94; 93.00(84.05-95.50); N=10 | 0.6642 | CpGI shore |
| SEMA3C_M2 |  |  | 59.28 ± 27.25; 62.83(33.92-78.58);N=6 | 61.07 ± 15.71; 64.83(52.83-72.00); N=10 | 0.9578 | CpGI |
| SLC2A10_M1 | chr20:45338279  -45364985 | NM_03  0777 | 88.50 ± 3.78; 88.60(84.65-92.30);N=6 | 85.93 ± 10.98; 87.60(78.80-94.20); N=9 | 0.9546 | CpGI shore |
| SLC2A10_M2 |  |  | 81.79 ± 8.84; 82.75(76.25-88.65);N=6 | 70.81 ± 8.04; 72.38(67.00-76.13); N=9 | 0.0256* | CpGI |
| SMAD6_M1 | chr15:66994674  -67074337 | NM_00  5585 | 14.83 ±6.71;  16.18(7.31-19.75); N=6 | 14.38 ± 3.73; 14.35(11.27-16.84); N=10 | 0.8749 | CpGI |
| SMAD6_M2 |  |  | 7.69 ± 2.57;  6.56(5.92-10.03); N=5 | 6.77 ± 1.44;  6.34(5.78-7.24); N=8 | 0.5576 | CpGI |
| SOX11 | chr2:5832799  -5841517 | NM_00  3108 | 31.96 ±15.77; 37.19(18.56-44.34);N=6 | 34.17 ± 17.09; 39.04(23.31-48.78); N=10 | 0.6354 | CpGI |
| SOX4 | chr6:21593972  -21598849 | NM_00  3107 | 92.57 ± 1.65; 92.60(91.70-94.05);N=6 | 92.11 ± 5.84; 93.60(90.70-95.40); N=9 | 0.4787 | CpGI shore |
| TBX1_M1 | chr22:19744226  -19767068 | NM_08  0646 | 12.84 ± 5.77;  13.70(7.40-17.85); N=5 | 12.10 ± 4.68;  11.36(8.82-15.62); N=9 | 0.7972 | CpGI |
| TBX1_M2 |  |  | 8.78 ± 4.34;  9.00(4.33-13.00); N=3 | 9.04 ± 3.70;  8.39(6.03-12.58); N=8 | 1 | CpGI |
| TBX5_M1 | chr12:114791735  -114846247 | NM_08  0717 | 42.16 ± 14.67; 42.04(32.97-54.69);N=6 | 64.40 ± 9.83; 63.31(55.70-74..56); N=10 | 0.0047* | CpGI shore |
| TBX5_M2 |  |  | 20.97 ± 26.29;  10.03(8.04-30.33); N=6 | 8.26 ± 3.26; 8.56(5.20-11.03);N=10 | 0.2198 | CpGI |
| TBX10 | chr11:67398774  -67407031 | NM_00  5995 | 69.98 ± 4.59; 69.67(65.70-74.56);N=4 | 72.52 ± 11.98; 73.25(69.33-80.42); N=7 | 0.5273 | TSS |
| TBX20_M1 | chr7:35271112  -35293711 | NM_00  1166220 | 38.19 ± 7.85; 38.73(30.63-45.47);N=5 | 14.33 ± 7.54;  12.60(8.83-17.77); N=8 | 0.0031** | CpGI |
| TBX20_M2 |  |  | 4.98 ± 1.08;  5.19(3.78-5.88); N=6 | 5.20 ± 1.57;  4.91(4.02-6.38); N=10 | 0.7925 | CpGI |
| TEAD1 | chr11:12695969  -12966284 | NM_02  1961 | 7.34 ± 1.98;  6.47(6.03-9.56); N=6 | 5.94 ± 1.89;  5.40(4.40-7.93); N=10 | 0.0934 | CpGI |
| TEK | chr9:27109147  -27230172 | NM_00  0459 | 29.46 ± 9.58; 25.20(21.83-39.22);N=5 | 43.56 ± 12.56; 41.40(34.75-48.40); N=9 | 0.0420* | TSS |
| TGFB2 | chr1:218518676  -218617961 | NM_00  3238 | 7.34 ± 1.29;  7.27(6.09-8.67); N=3 | 8.53 ± 5.52;  8.25(2.86-12.68); N=9 | 0.8636 | CpGI |
| TGFBR3_M1 | chr1:92145900  -92351836 | NM_00  3243 | 91.60 ± 5.00; 91.80(86.50-96.50);N=3 | 84.08 ± 10.71; 89.75(81.29-91.00); N=7 | 0.2667 | CpGI shore |
| TGFBR3_M2 |  |  | 33.39 ± 18.10; 35.17(15.22-49.78);N=4 | 20.13 ± 8.70; 17.78(15.62-22.81); N=10 | 0.2398 | CpGI |
| TLL1_M1 | chr4:166794410  -166952111 | NM_00  1204760 | 26.94 ± 7.84; 26.91(20.98-35.07);N=6 | 28.04 ± 9.80; 27.28(21.75-31.94); N=10 | 0.9578 | CpGI shore |
| TLL1_M2 |  |  | 11.20 ± 3.23;  12.19(7.41-13.92); N=6 | 11.76 ± 4.81;  12.37(7.34-13.61); N=10 | 0.786 | CpGI |
| VCAM1 | chr1:101185196  -101204601 | NM_08  0682 | 21.00 ± 7.31; 21.00(14.13-28.00);N=6 | 19.88 ± 7.15; 20.13(12.25-26.44); N=10 | 0.5502 | TSS |
| ZFPM2 | chr8:106331147  -106816767 | NM_01  2082 | 59.63 ± 18.55; 59.63(44.79-73.83);N=6 | 83.07 ± 6.07; 83.00(78.50-85.42); N=9 | 0.0256* | CpGI shore |
| ZIC3_M1 | chrX:136648346  -136654259 | NM_00  3413 | 22.19 ±18.94;  13.17(9.92-37.63); N=6 | 16.76 ± 7.70; 15.83(9.67-23.08); N=9 | 0.8639 | CpGI shore |
| ZIC3_M2 |  |  | 18.23 ± 15.31;  17.97(4.33-32.4); N=4 | 20.17 ± 23.92;  5.81(3.40-47.13); N=7 | 0.6485 | CpGI |
| FKBP1A | chr20:1349621  -1373816 | NM_00  1199786 | 8.44 ±1.75;  8.15(6.77-10.39); N=6 | 9.83 ± 3.47;  8.71(7.54-11.50); N=10 | 0.5149 | CpGI |
| PSEN1_M1 | chr14:73603143  -73690399 | NM_00  7318 | 87.06 ± 12.32; 92.64(80.53-93.64);N=6 | 91.41 ± 3.52; 92.00(90.00-94.00); N=10 | 0.7925 | CpGI shore |
| PSEN1_M2 |  |  | 86.63 ± 2.53; 87.40(85.65-88.10),N=6 | 84.14 ± 3.71; 83.20(82.55-87.00); N=10 | 0.2119 | CpGI |
| PSEN1_M3 |  |  | 9.72 ± 1.89; 9.33(7.97-11.92);N=6 | 8.50 ±2.00;  8.07(6.77-10.43); N=10 | 0.2198 | CpGI |

**Additional file 1: Table S5．The median methylation levels of 26 candidate genes showing significant difference in TOF cases and controls**

| Gene | Genebank accession | Gene  amplicon | Control,  median (IQR), N | TOF ,  median(IQR), N | P value* | Position |
| --- | --- | --- | --- | --- | --- | --- |
| CFC1B | NM_001079530 | CFC1B_M2 | 48.16(40.36-59.25);N=6 | 67.71(54.67-80.67);N=10 | 0.0375* | TSS |
| DVL2 | NM_004422 | DVL2_M2 | 9.09(6.81-11.22); N=6 | 12.44(11.45-14.30); N=9 | 0.0176* | CpGI |
| EDN1 | NM_001168319 | EDN1_M2 | 11.46(8.29-14.06); N=5 | 7.29(6.38-8.96); N=9 | 0.0290* | CpGI |
| EGFR | NM_201282 | EGFR_M1 | 52.75(44.00-56.35);N=6 | 59.17(55.57-64.92);N=10 | 0.040* | CpGI |
| EDNRA | NM_001957 | EDNRA | 42.25(34.69-44.42); N=6 | 46.13(40.63-50.88); N=9 | 0.049* | CpGI shore |
| EVC2 | NM_001166136 | EVC2_M1 | 29.63(18.84-32.25);N=6 | 36.38(31.88-48.81); N=9 | 0.0190* | CpGI |
| GJA5 | NM_005266 | GJA5 | 32.83(29.00-36.08);N=6 | 48.33(44.25-54.00);N=10 | 0.0225* | TSS |
| HAND1 | NM_004821 | HAND1_M1 | 12.71(6.73-24.48); N=4 | 30.77(21.59-51.65);N=10 | 0.0360* | CpGI |
| HAS2 | NM_005328 | HAS2_M2 | 3.75(3.31-6.65); N=6 | 7.68(5.78-11.38); N=8 | 0.0200* | CpGI |
| HOXA3 | NM_030661 | HOXA3 | 47.33(38.44-52.56); N=5 | 31.17(26.33-37.78); N=8 | 0.0190* | TSS |
| HSPG2 | NM_005529 | HSPG2_M2 | 3.75(3.31-6.65); N=6 | 7.68(5.78-11.38); N=8 | 0.0200* | CpGI |
| MED13L | NM_015335 | MED13L_M1 | 82.57(76.69-86.17); N=5 | 90.94(85.64-91.78);N=10 | 0.0400* | CpGI shore |
| MYH6 | NM_002471 | MYH6_M1 | 39.00(30.63-46.25); N=6 | 18.25(7.38-36.25); N=14 | 0.0355* | TSS |
| NFATC1 | NM_172389 | NFATC1_M1 | 84.79(80.43-90.59); N=4 | 93.00(88.25-95.75); N=9 | 0.0503* | CpGI shore |
| NKX2-5 | NM_001166175 | NKX2-5 | 22.72(20.46- 24.83); N=4 | 33.38(30.91-51.49); N=8 | 0.0081** | CpGI |
| NR2F2 | NM_021005 | NR2F2 | 62.50(54.35-68.30);N=6 | 45.00(34.30-53.80);N=10 | 0.0312* | CpGI shore |
| NRG1 | NM_001160004 | NRG1 | 24.00(16.00-28.25); N=4 | 9.50(6.00-15.75); N=9 | 0.0196* | CpGI |
| NRP1 | NM_003873 | NRP1_M1 | 19.29±15.66;  12.00(8.43-33.79); N=5 | 5.80 ± 3.07;  5.57(2.75-8.79); N=10 | 0.0127* | CpGI shore |
| NFATC2 | NM_001136021 | NFATC2_M2 | 21.80(17.50-25.53);N=6 | 30.39(24.91-42.56);N=10 | 0.0252* | CpGI |
| PAX3 | NM_181459 | PAX3_M2 | 3.25(2.89-4.99); N=6 | 5.64(4.05-8.86); N=10 | 0.0392* | CpGI |
| PDGFRA | NM_006206 | PDGFRA_M2 | 18.31(16.23-32.22); N=6 | 14.75(5.88-16.63); N=9 | 0.0337* | CpGI |
| SLC2A10 | NM_030777 | SLC2A10_M2 | 82.75(76.25- 88.65); N=6 | 72.38(67.00-76.13); N=9 | 0.0256* | CpGI |
| TBX5 | NM_080717 | TBX5_M1 | 42.04(32.97-54.69); N=6 | 63.31(55.70-74..56);N=10 | 0.0047** | CpGI shore |
| TBX20 | NM_001166220 | TBX20_M1 | 38.73(30.63-45.47); N=5 | 12.60(8.83-17.77); N=8 | 0.0031** | CpGI |
| TEK | NM_000459 | TEK | 25.20(21.83-39.22); N=5 | 41.40(34.75-48.40); N=9 | 0.0420* | TSS |
| ZFPM2 | NM_012082 | ZFPM2 | 59.63(44.79-73.83); N=6 | 83.00(78.50-85.42); N=9 | 0.0256* | CpGI shore |

**P* < 0.05, ***P* < 0.01, ****P* < 0.001 (Mann-Whitney test)；IQR，interquartile range.
